# Copper coordination polymers from cavitand ligands: hierarchical spaces from cage and capsule motifs, and other topologies†
†Electronic supplementary information (ESI) available: Further details of crystallographic studies, thermogravimetric analyses, Raman spectroscopy. CCDC 1401250–1401256. For ESI and crystallographic data in CIF or other electronic format see DOI: 10.1039/c5sc01801c



**DOI:** 10.1039/c5sc01801c

**Published:** 2015-07-14

**Authors:** Flora L. Thorp-Greenwood, Tanya K. Ronson, Michaele J. Hardie

**Affiliations:** a School of Chemistry , University of Leeds , Woodhouse Lane , Leeds LS2 9JT , UK . Email: m.j.hardie@leeds.ac.uk

## Abstract

The cyclotriveratrylene-type ligands (±)-tris(iso-nicotinoyl)cyclotriguaiacylene L1 (±)-tris(4-pyridylmethyl)cyclotriguaiacylene L2 and (±)-tris{4-(4-pyridyl)benzyl}cyclotriguaiacylene L3 all feature 4-pyridyl donor groups and all form coordination polymers with Cu^I^ and/or Cu^II^ cations that show a remarkable range of framework topologies and structures. Complex [Cu^I^_4_Cu^II^_1.5_(L1)_3_(CN)_6_]·CN·*n*(DMF) **1** features a novel 3,4-connected framework of cyano-linked hexagonal metallo-cages. In complexes [Cu_3_(L2)_4_(H_2_O)_3_]·6(OTf)·*n*(DMSO) **2** and [Cu_2_(L3)_2_Br_2_(H_2_O)(DMSO)]·2Br·*n*(DMSO) **3** capsule-like metallo-cryptophane motifs are formed which linked through their metal vertices into a hexagonal 2D network of (4^3^.12^3^)(4^2^.12^2^) topology or a coordination chain. Complex [Cu_2_(L1)_2_(OTf)_2_(NMP)_2_(H_2_O)_2_]·2(OTf)·2NMP **4** has an interpenetrating 2D 3,4-connected framework of (4.6^2^.8)(6^2^.8)(4.6^2^.8^2^) topology with tubular channels. Complex [Cu(L1)(NCMe)]·BF_4_·2(CH_3_CN)·H_2_O **5** features a 2D network of 6^3^ topology while the Cu^II^ analogue [Cu_2_(L1)_2_(NMP)(H_2_O)]·4BF_4_·12NMP·1.5H_2_O **6** has an interpenetrating (10,3)-*b* type structure and complex [Cu_2_(L2)_2_Br_3_(DMSO)]·Br·*n*(DMSO) **7** has a 2D network of 4.8^2^ topology. Strategies for formation of coordination polymers with hierarchical spaces emerge in this work and complex **2** is shown to absorb fullerene-C_60_ through soaking the crystals in a toluene solution.

## Abstract 2

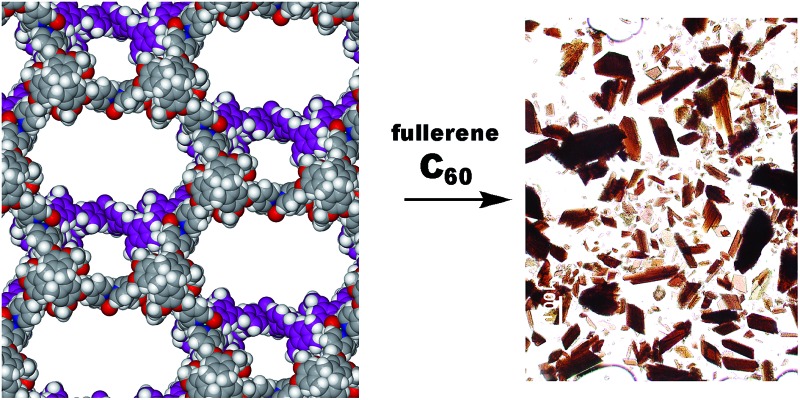
Copper coordination polymers from cavitand ligands are reported including networked cage-motif structures, one of which takes up C_60_ from solution.

## Introduction

Coordination polymers and metal–organic frameworks (MOFs) are crystalline coordination compounds with infinite framework structures constructed from metal cations and bridging ligands.^1^ Potential applications for coordination polymers have been demonstrated or proposed in a myriad of fields including magnetism, non-linear optics, catalysis, separations and extractions, and gas storage.^1^ Many of these applications are dependent on the ability of coordination polymers or MOFs to bind guest molecules, and materials which feature a robust, porous structure which is maintained on evacuation of mother liquor are particularly prominent. Coordination polymer materials that feature channels or cavities in their framework but are not robust to loss of all guest solvent may also find function as heterogeneous hosts provided guest molecules can be exchanged without substantial loss of framework structure. This principle has recently been used to great effect by Fuijta and others who have developed a crystalline-sponge approach for the determination of otherwise inaccessible crystal structures of guest molecules exchanged into the cavities of coordination polymer hosts.^2^

The ability of coordination polymers to function as a host is a property of the overall assembly and not of the individual molecular or ionic building blocks that make up the framework. Molecules that have an intrinsic ability to bind guest molecules are also well known and are referred to as molecular hosts, and most examples are cyclic in nature. The use of molecular hosts as components of coordination polymers may result in a material which contains both the specific molecular recognition sites of the molecular host and channels and cavities of the coordination polymer framework. As such, they can be regarded as materials with a hierarchical pore structure. Cyclodextrins, for example, form robust metal–organic frameworks which have applications in gold extraction and CO_2_ capture.^3^ Other well-known classes of molecular hosts including calixarenes and related cone-conformation hosts, and cucurbiturils have also been reported to form coordination polymers, often when suitably functionalised with metal-binding groups.^4–6^ Cyclotriveratrylene (CTV) is a bowl-shaped molecular host with a tribenzo[*a*,*d*,*g*]cyclononatriene core. CTV forms chain and 2D coordination polymers, most commonly with group 1 metal cations.^7^ CTV analogues, including the demethylated cyclotricatechylene^8^ and a range of tripodal ligand-functionalised analogues of CTV^9–15^ have been shown to form coordination polymers that contain embedded molecular hosts. Some of these coordination polymer materials exhibit structures with large channels or cavities and where the molecular bowl of the tribenzo[*a*,*d*,*g*]cyclononatriene ligand scaffolds are potentially accessible to new guest molecules.^8–10^ In the majority of examples, however, the molecular recognition sites are not accessible due to inter- or intra-network host–guest interactions. These include bowl-in-bowl stacking motifs between the tribenzo[*a*,*d*,*g*]cyclononatriene scaffolds,^11^ and a pronounced tendency for the formation of self-complementary interactions between two CTV-type ligands to form a dimeric so-called hand-shake motif, or through inter-network host–guest interactions between a terminal additional ligand group and the tribenzo[*a*,*d*,*g*]cyclononatriene host core.^10,12^ 
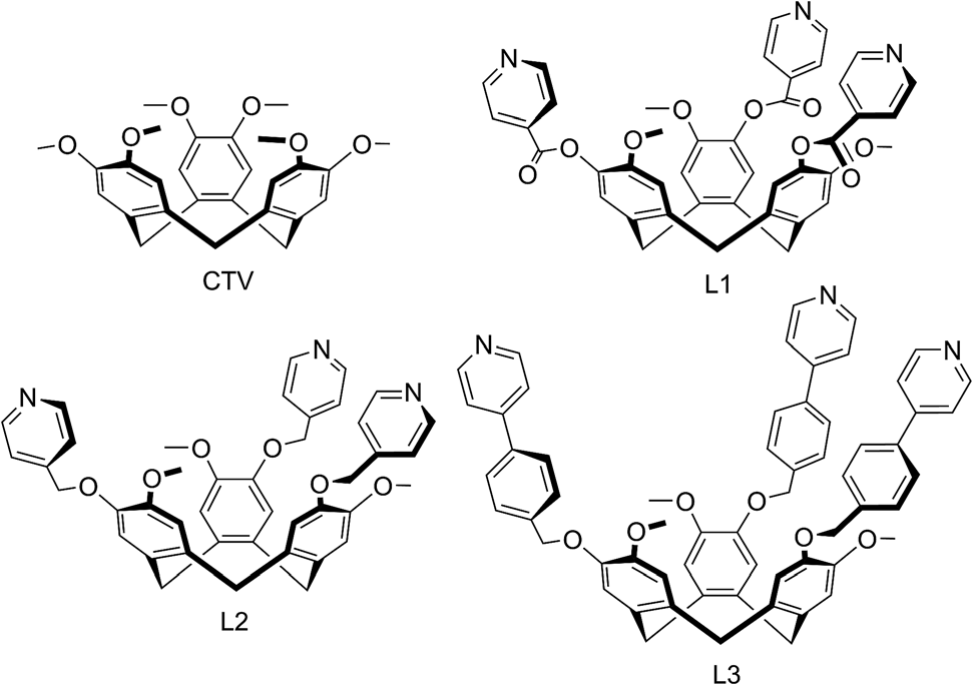


We report here a series of new coordination polymer materials that have been accessed through the combination of copper cations and CTV-type ligands where the tribenzo[*a*,*d*,*g*]cyclononatriene core has been decorated with a tripodal arrangement of 4-pyridyl ligand groups, namely (±)-tris(iso-nicotinoyl)cyclotriguaiacylene L1,^15^ (±)tris(4-pyridylmethyl)cyclotriguaiacylene L2 (ref. 16) and (±)tris{4-(4-pyridyl)benzyl}cyclotriguaiacylene L3,^17^ all of which we have previously reported. Nominally *C*_3_-symmetric tripodal derivatives are chiral, and we utilise them as racemic mixtures. Only the iso-nicotinoyl appended L1 has been previously shown to form a coordination polymer in a chain structure with Ag(i),^15^ however all three ligands are known to form discrete metallo-supramolecular species.^17–19^ Complexes with ligand L1 have been the most studied and include Pd_6_L_8_ stella octangula cage species,^17,18^ capsule-like metallo-cryptophane Pd_3_L_2_ species,^19^ and a Cu_6_L_6_ metallacycle formed from CuBr_2_ that has a topologically unique Borromean-like chainmail arrangement.^20^ The materials reported here represent a remarkable structural variation and include unusual topologies induced by the distinctive pyramidal shape of these ligands, coordination polymers incorporating cage or capsule-motifs with both large lattice channels and accessible molecular recognition sites, as well as new examples of more common topological types.

## Results and discussion

### Coordination polymers with cage and capsule motifs

Reaction of L1 and CuCN in dimethylformamide (DMF) initially results in formation of a pale green viscous solution out of which small red-orange single crystals grow after approximately 3 weeks of standing. The red-orange crystals were of a 3D coordination polymer complex of composition [Cu^I^_4_Cu^II^_1.5_(L1)_3_(CN)_6_]·CN·*n*(DMF) **1**. The crystal structure indicates that there was air oxidation of some copper sites from Cu^I^ to Cu^II^. Interestingly, we also obtained complex **1** in small quantities from reaction of L1 in DMF with Cu^II^(NO_3_)_2_. The cyanide in the material obtained from Cu(NO_3_)_2_ is likely to have resulted from decomposition of the solvent, and it is notable that the IR spectrum of this material did not indicate the presence of any nitrate. Formation of copper cyanide based coordination polymers where the cyanide has come from reagent decomposition has been reported before from cleavage of diaminomaleonitrile.^21^ Mixed valence copper coordination polymers have also been previously reported, although examples are not numerous.^22^

**Fig. 1:**
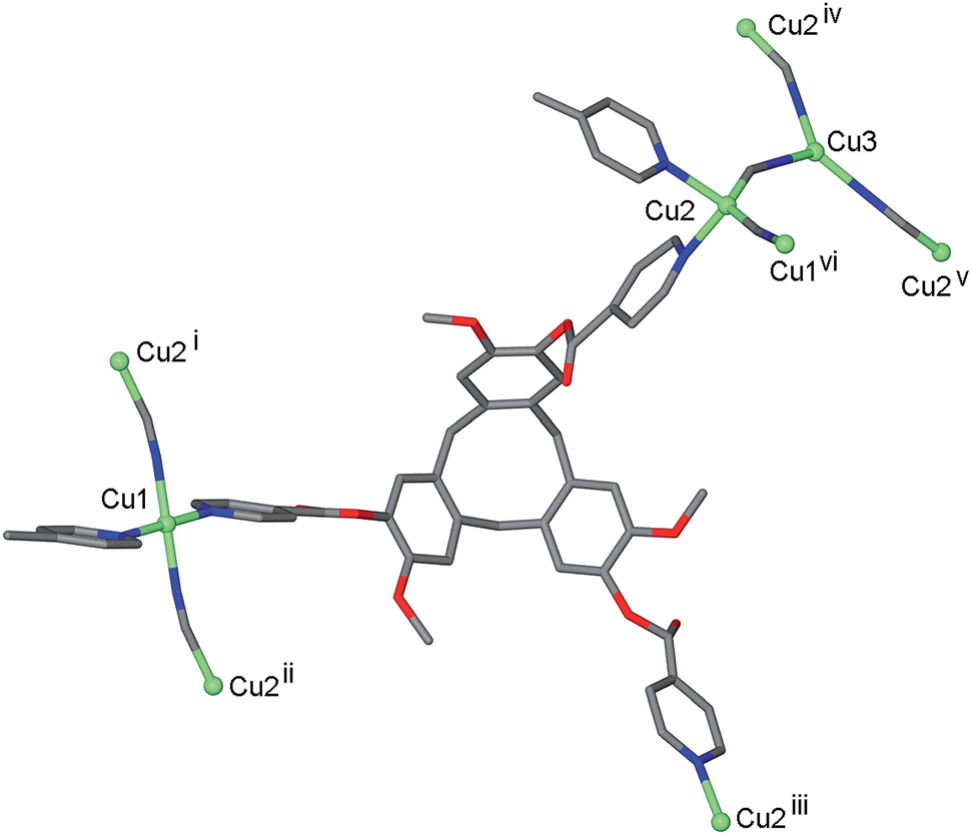
Coordination geometries of Cu sites and ligand bridging behaviour from the crystal structure of complex **1**. Symmetry operators: (i) *y*, –*x* + *y*, 1 – *z*; (ii) 2/3 – *y*, 1/3 + *x* – *y*, 1/3 + *z*; (iii) *x* – *y*, *x*, 1 – *z*; (iv) –*x* + *y*, 1 – *x*, *z*; (v) 1 – *y*, 1 + *x* – *y*, *z*; (vi) 1/3 – *x* + *y*, 2/3 – *x*, *z* – 1/3.

The infrared spectrum of complex **1** indicates the presence of cyanide with a very broad peak centred at 2127 cm^–1^. The single crystal structure was determined in the space group *R*3, with only the [Cu^I^_4_Cu^II^_1.5_(L1)_3_(CN)_6_]^+^ framework able to be structurally elucidated, counter-anions and any included solvent were not located due to disorder and subsequent weakly diffracting nature of the material. There are three crystallographically distinct Cu sites: a square planar Cu^II^ sited on an inversion centre (Cu1), a tetrahedral Cu^I^ on a general position (Cu2), and a trigonal Cu^I^ on a three-fold axis (Cu3), Fig. 1. Oxidation states were assigned according to expected coordination behaviour of copper, noting that there have been a number of precedents for trigonal planar Cu^I^ with bridging cyanide ligands within coordination polymer materials.^23^

Square planar is not a common geometry for Cu^I^ in an unconstrained ligand environment such as this one and the bond valence sum calculation^24^ for this cation was also consistent with Cu^II^. Each square planar Cu1 site is coordinated by *trans* cyanide and *trans* pyridyl groups from two L1 ligands, each Cu2 site is coordinated by the same ligand set but in tetrahedral geometry and each Cu3 is coordinated by three CN– ligands. Cyanide bridges occur between Cu1 and Cu2 sites, and between Cu2 and Cu3 sites, Fig. 1, with Cu··· Cu distances between 4.85 and 4.87 Å. The tripodal ligand L1 deviates from molecular *C*_3_-symmetry with one isonicotinoyl group rotated such that the ester carbonyl is sited above the molecular cavity of the CTV-scaffold, and coordinates to Cu centres through all three pyridyl-donors bridging between Cu1 and Cu2 type centres, Fig. 1.

**Fig. 2:**
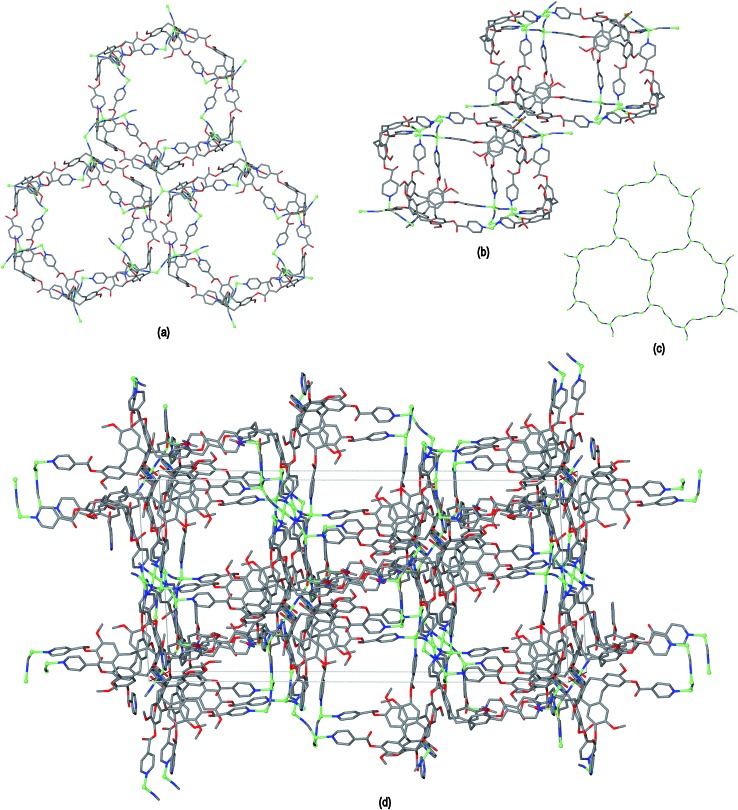
Crystal structure of complex **1** illustrating the formation of hexagonal prism motifs within the 3D coordination polymer framework, (a) top view down the *c* axis of three prisms connected by a central Cu3 ion; (b) side view of two prisms from two different layers connected through Cu–CN bridges; (c) the copper cyanide expanded hexagonal net; (d) unit cell diagram viewed down the *a* axis.

**Fig. 3:**
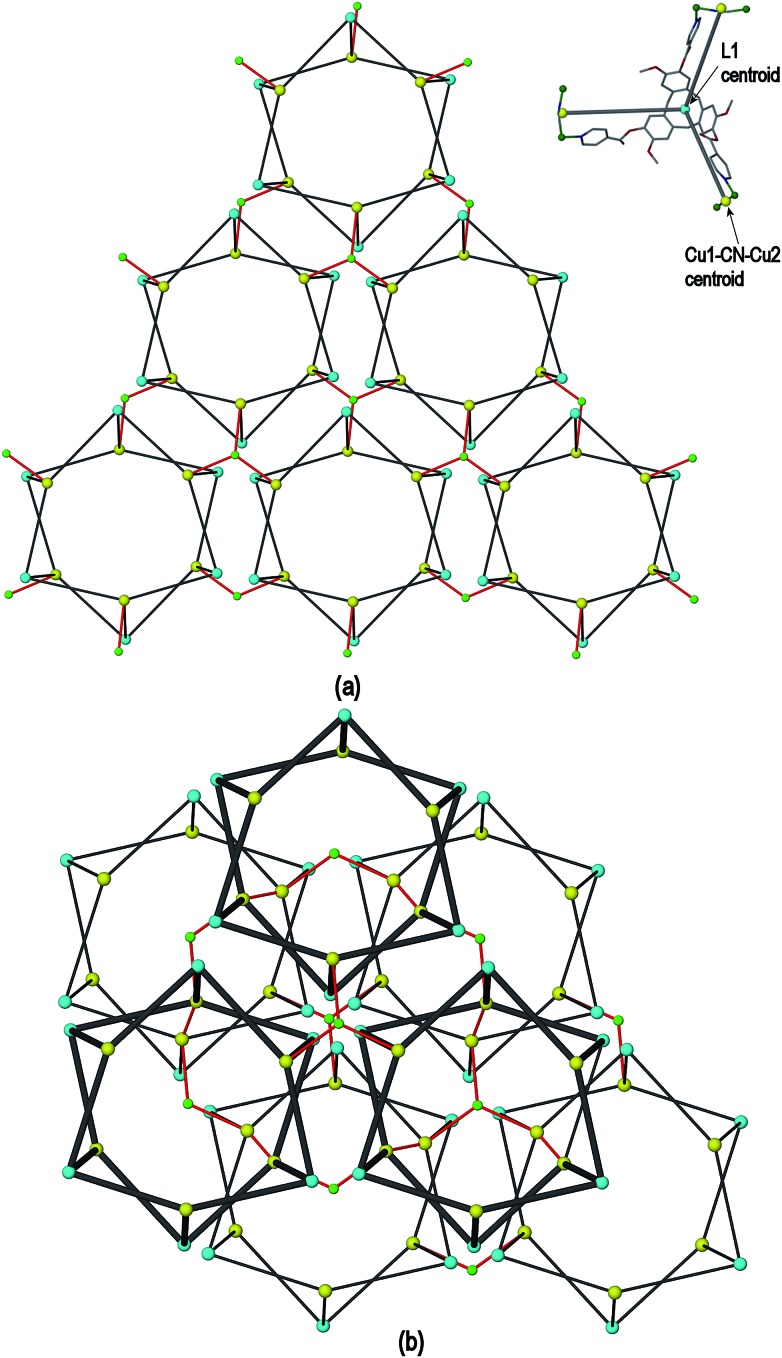
Simplified connectivity diagrams for complex **1** showing (a) one layer of prisms connected by Cu3 centres; (b) two layers with upper layer in heavier lines illustrating the close-packing relationship between the prisms. Linkages between prisms are shown in red, green spheres are Cu3 positions, blue spheres represent L1 ligands and yellow spheres centres of Cu1–CN–Cu2 linkages as shown upper right of Figure.

The resultant [Cu^I^_4_Cu^II^_1.5_(L1)_3_(CN)_6_]^+^ coordination polymer has an unusual 3,4-connected 3D framework structure. A marked feature of the framework is the formation of hexagonal prismatic cages bounded by Cu1 and Cu2 centres, cyanides and six L1 ligands, Fig. 2. The six L1 ligands occupy half the vertices of the hexagonal prisms, alternating with a prism vertex involving a Cu1–(CN)–Cu2 motif. All have the molecular bowls of the L1 ligands oriented inwards to create the prismatic cage, and each cage contains three of each ligand L1 enantiomer. The distance across the diagonal of the prism taken between L1 centroids (see Fig. 3 for definition) is *ca*. 3.1 nm. The hexagonal prisms are linked into a 2D network in the *ab* plane through cyanide bridges to the trigonal planar Cu3, Fig. 2(a), and offset layers of prisms are connected together through further copper cyanide bridges, Fig. 2(b). The unit cell packing diagram viewed down the *a* axis is shown in Fig. 2(d) and illustrates that channels run throughout the structure. The 3D coordination polymer framework can also be considered as being composed of copper-cyanide expanded hexagonal layers running in the *ab* plane that are linked together through bridging L1 ligands. Each copper-cyanide ring in the hexagonal layer has a Cu_24_(CN)_24_ composition, Fig. 2(c).

A simplified connectivity diagram of the 3D network of complex **1** is shown in Fig. 3, where the Cu1–CN–Cu2 linkages within a prism are taken as a single connecting centre and shown as yellow spheres. These diagrams illustrate how the hexagonal prisms within **1** are connected together in a manner which mimics the close packing of spheres. Fig. 3(a) shows a single close-packed layer of prisms linked through Cu3 centres (green spheres). A second layer, shown in heavier lines in Fig. 3(b), is oriented above the first in a manner which creates both octahedral and tetrahedral sites as would be seen in close packing of spheres. The overall layer packing pattern is ABC.

Thermogravimetric analysis (TGA) of crystals of **1** that were collected in air but not evacuated under vacuum indicates a weight loss of *ca.* 10 % to 250 °C after which the material undergoes significant weight loss to 320 °C, the latter process indicating decomposition. The initial weight loss is due to solvent loss and corresponds to approximately 4 molecules of DMF per formula unit. The crystals show rapid deterioration after removal from mother liquor, and the level of solvation is likely to be higher when the material is kept under DMF, noting the [Cu^I^_4_Cu^II^_1.5_(L1)_3_(CN)_6_]^+^ framework of **1** accounts for less than 40% of the crystal volume.

**Fig. 4:**
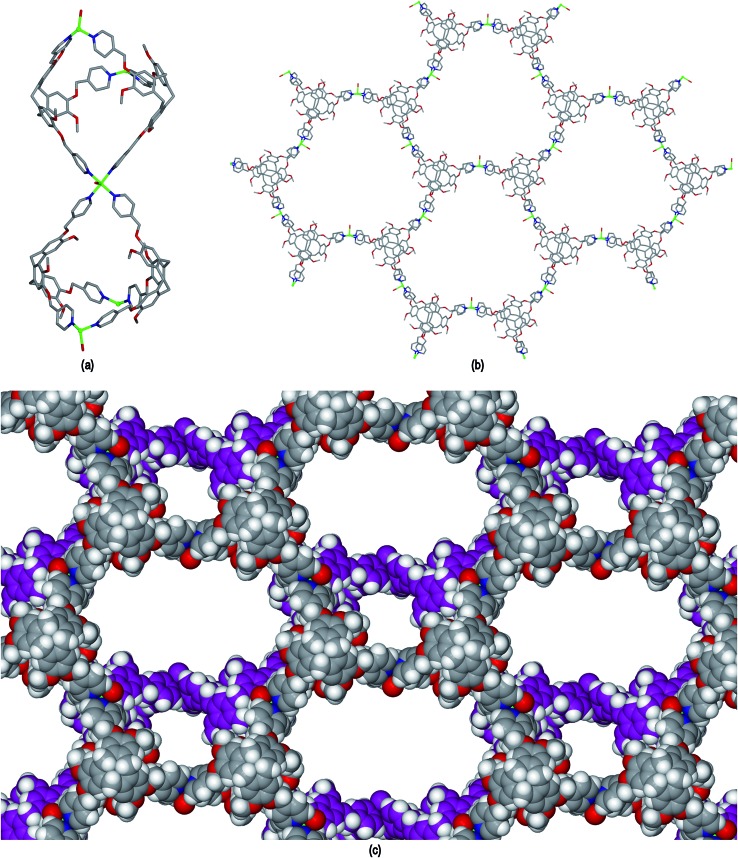
From the crystal structure of [Cu_3_(L2)_4_(H_2_O)_3_]·6(OTf) **2**. (a) The metallo-cryptophanes of the asymmetric unit; (b) chiral 2D network of vertice-linked metallo-cryptophanes that forms in *bc* plane; (c) stacking of two layers of enantiomorphic 2D metallo-cryptophane layers shown in space-filling mode and in different colours for clarity.

The ligand L2 differs from L1 only in having a more conformationally flexible methyl ether linkage rather than ester between the pyridyl group and CTV scaffold, however the coordination polymers obtained with L2 are markedly different to those from L1. Plate-like lilac-coloured crystals of complex [Cu_3_(L2)_4_(H_2_O)_3_]·6(OTf)·*n*(DMSO) **2** were obtained from the reaction of Cu(OTf)_2_ and L2 in dimethylsulfoxide (DMSO). The crystal structure was solved in space group *C*2/*c*. The given formula represents double the asymmetric unit of the structure, although counter-anions and any solvent were not located due to disorder. One crystallographically distinct Cu^II^ centre is sited on a two-fold axis whilst the other is on a general position. Each have similar square pyramidal coordination geometries being coordinated by an aquo ligand in the apical sites (Cu–O distances 2.337(4) to 2.407(8) Å) and pyridyl groups of L2 ligands in the four basal positions (Cu–N distances 1.975(6) to 2.047(5) Å). Each L2 ligand bridges between three Cu^II^ centres and both crystallographically distinct L2 ligands have similar conformations where each pyridyl group is approximately coplanar with its associated scaffold phenyl group. This is very much in contrast to the structures of complex **1** (and indeed the other complexes of L1 reported here) where the pyridyl (of iso-nicotinoyl) groups were closer to orthogonal with adjoining scaffold phenyls. The L2 ligands are arranged into head-to-head pairs linked together through the Cu^II^ cations to give a capsule-like motif, Fig. 4(a). Organic capsules formed from the head-to-head dimerization of CTV fragments are known as cryptophanes, and may form as chirally pure *anti* isomers or the *meso*–*syn* isomer.^25^ Capsule species where two CTV-ligands are bound together in a M_3_L_2_ species are referred to as metallo-cryptophanes, and discrete metallo-cryptophanes are known with 4-pyridyl-decorated CTV ligands.^19,26^ The closest Cu···Cu separations within the metallo-cryptophane motif of complex **2** are of the order of 17–17.5 Å and the distance between L2 centroids is 15.1 Å. Each Cu^II^ centre is shared by two metallo-cryptophanes to form a 2D metallo-cryptophane network with large hexagonal cavities, Fig. 4(b). This is a highly unusual 3,4-connected network with (4^3^.12^3^)(4^2^.12^2^) topology. Each cavity within the network is bound by six cryptophanes and six Cu^II^ centres with three aquo ligands directed into the cavity. The longest Cu···Cu separation across the cavity is 34.1 Å and the distance from the centre of the cavity to the O atom of an aquo ligand is 12.1 Å.

The 2D networks form in the *bc* plane and stack together along the *a* axis in an AB arrangement. The phenyl groups of one network are roughly aligned with pyridyls of the adjacent network with ring centroid distances between them ranging from 4.03 to 4.59 Å indicating any π–π stacking interactions in the lattice are weak. The 2D networks are not perfectly aligned, but show a displacement which creates two types of channel when viewed down the *a* axis: a smaller channel of approximately 8 Å cross-section and a larger channel with a cross-section of approximately 15 × 30 Å, Fig. 4(c). Each L2 ligand within a single 2D network is of the same enantiomer, thus forming *anti*-type metallo-cryptophanes and indicating chiral self-sorting occurs during the formation of each network in complex **2**. Overall, the complex is not chiral, as crystals contain an equal number of networks containing each ligand enantiomer which alternate along *a*.

A coordination polymer with embedded organic cryptophanes has been reported,^27^ as have coordination networks of metallo-capsules from pyrogallol[4]arenes, calix[4]arenes or cucurbiturils.^4^ We have previously reported a 2D network of linked metallo-cryptophanes in the complex [Ag_3_(NCMe)_3_(L)_2_Cl]^2+^ where L = tris{4-(3-pyridyl)phenylester}cyclotriguaiacylene.^14^ In that case the Ag_3_L_2_ metallo-cryptophanes were linked through Ag-*μ*_3_-Cl-Ag bridges, and there were no significant channels through the structure due to the manner of packing between the 2D networks. Robson's cyclotricatechylene-based coordination polymer features tetrahedral cages linked together into a network through oxide bridges.^8^ A number of metallo-cage motifs within metal–organic frameworks are known,^28^ and include Fujita's coordination polymer comprised of vertex-linked octahedral cage assemblies, materials which have recently been shown to act as crystalline sponges.^2,29^

**Fig. 5:**
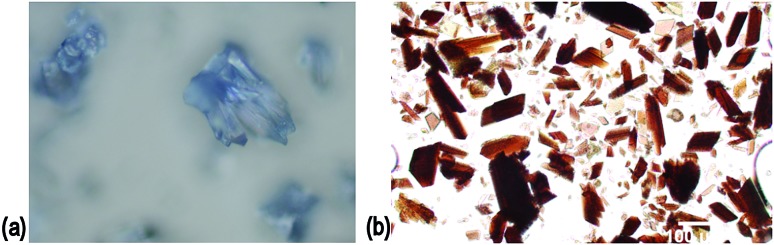
(a) Crystals of complex **2** as synthesised; (b) after soaking in toluene solution of fullerene-C_60_, crystal clusters have been broken up.

Complex **2** is not a robust material and loses crystallinity on loss of solvent. TGA indicates that the lattice contains at least five additional molecules of DMSO per formula unit that were not located in the crystal structure (mass loss *ca.* 9% to 250 °C, ESI†). The crystals are not deeply coloured hence are suitable candidates for initial experiments to determine whether **2** can act as a crystalline sponge. The mother liquor was decanted from a batch of complex **2** single crystals and rapidly replaced with a toluene solution of fullerene-C_60_. After soaking at ambient conditions for two weeks, the crystals adopted a red-brown colouration, Fig. 5, which is apparent throughout the crystal and not a surface effect. Raman spectroscopy and microanalysis of the bulk sample were consistent with the presence of fullerene-C_60_ in the crystals (see ESI†). The unit cell was established to be the same as the parent crystals through X-ray diffraction. Unfortunately the complex **2**@C_60_ crystals did not diffract sufficiently well to allow for a full structure determination to be performed. Nevertheless, the ability of complex **2** to up-take large guests has been demonstrated.

**Fig. 6:**
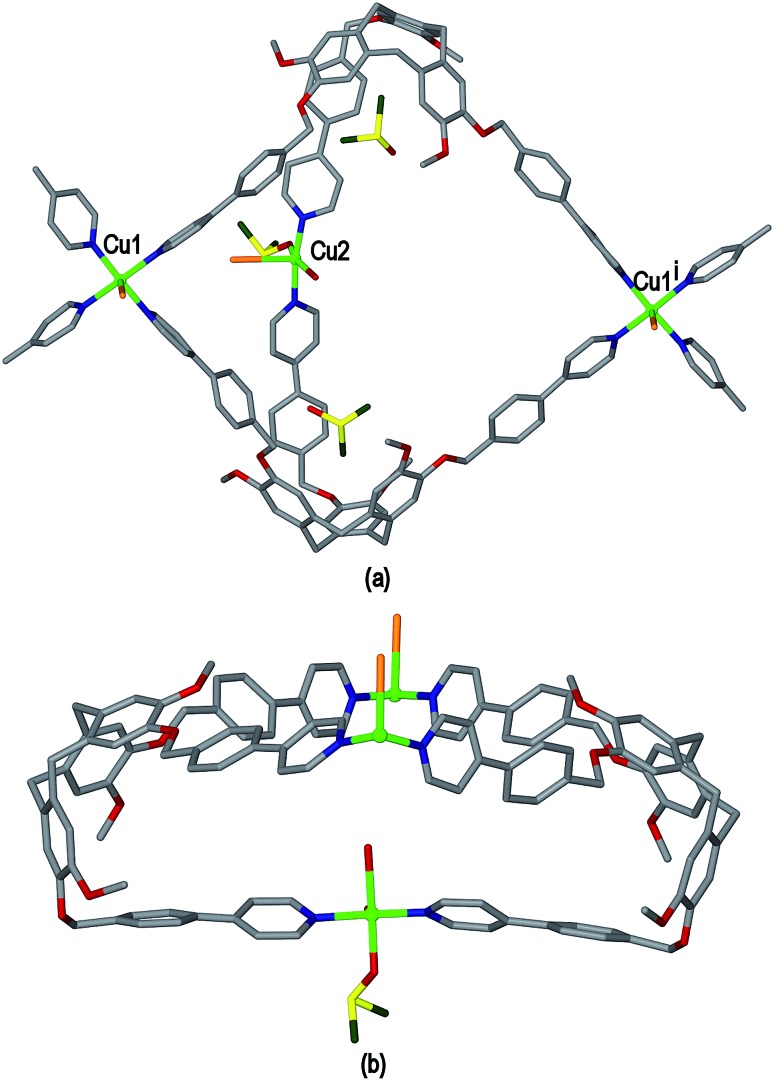
From the crystal structure of complex [Cu_2_(L3)_2_Br_2_(H_2_O)(DMSO)]·2Br·*n*(DMSO) **3**. (a) Single metallo-cryptophane moiety of the [Cu_2_(L3)_2_Br_2_(H_2_O)(DMSO)]^2+^ coordination chain with complete Cu^II^ coordination spheres shown and guest DMSO molecules; (ii) side-view of metallo-cryptophane with partial Cu1 coordination spheres shown for clarity. Symmetry element: (i) 1/2 + *x*, 3/2 – *y*, 1/2 + *z*.

Ligand L3 represents an extended-arm analogue of L2, and also forms a copper coordination polymer with linked metal-cryptophane structure. The complex [Cu_2_(L3)_2_Br_2_(H_2_O)(DMSO)]·2Br·*n*(DMSO) **3** forms from vapour diffusion of ethyl acetate into a DMSO solution of CuBr_2_ and L3. The crystal structure was solved in the space group *P*2_1_/*n* and the given formula represents the asymmetric unit, with the uncoordinated Br^–^ counter-anions modelled as disordered across several sites, and three molecules of DMSO were located in the structure. The two Cu^II^ centres both have square pyramidal geometry but have quite different coordination spheres. Cu1 features an axial Br^–^ (Cu–Br 2.707(3) Å) and is also coordinated by four pyridyl groups from four L3 ligands (Cu–N 2.003(11) to 2.054(10) Å), while Cu2 has an axial Br^–^ ligand (Cu–Br 2.793(5) Å), but only two *trans* pyridyl ligands (Cu–N 1.912(18) and 2.034(9) Å) with the remainder of the coordination sphere occupied by aquo and DMSO ligands, Fig. 6(a). The L3 ligands are linked in a head-to-head fashion to form achiral *syn* isomer metallo-cryptophanes, unlike in complex **3** where they were the chiral *anti* isomer. DMSO guest molecules were located for both L3 ligands, and are oriented with methyl groups directed towards the hydrophobic cavity, Fig. 6(a). The metallo-cryptophanes of complex **3** are also distinct in that they do not possess approximate *C*_3_-symmetry. For both crystallographically distinct L3 ligands, one of the methylene–phenyl–pyridyl side-arms is bent inwards over the cavitand ligand molecular bowl such that the C_aryl_–O–CH_2_–C_aryl_ torsion angle is *ca*. 75°, whereas the equivalent torsion angle is closer to 180° for the other methylene–phenyl-pyridyl side-arms. This means the three Cu^II^ centres that form the equatorial region of the metal-cryptophane form a scalene rather than equilateral triangle (Cu···Cu distances 23.9, 16.2, 11.8 Å), giving the metallo-cryptophane motif a distinctly flattened aspect, Fig. 6(b).

**Fig. 7:**
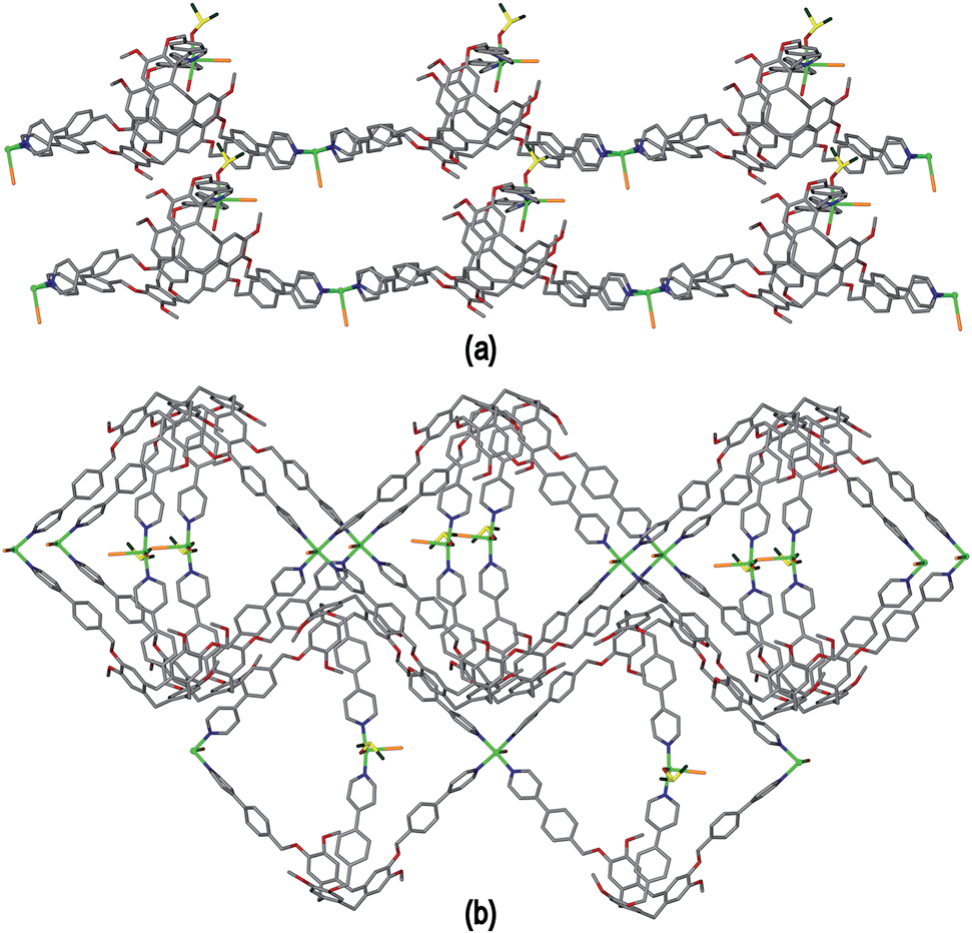
From the crystal structure of complex [Cu_2_(L3)_2_Br_2_(H_2_O)(DMSO)]·2Br·*n*(DMSO) **3**. (a) View of two [Cu_2_(L3)_2_Br_2_(H_2_O)(DMSO)]^2+^ coordination chains aligned in a p[olar fashion; (b) packing of coordination chains viewed down the *a* axis, solvent, hydrogen atoms and disordered counter-anions excluded for clarity.

As in complex **2**, linked metallo-cryptophane moieties are formed in **3**, however here the metallo-cryptophanes are linked through only two of three Cu^II^ vertices to form a chain rather than 2D network, noting it is Cu2 which is bound by the folded-in ligand arms which is the topologically-trivial link, Fig. 7(a). Each chain is polar with each metallo-cryptophane in the same orientation and they pack together in a polar fashion along the *a* unit cell, Fig. 7(a). The overall lattice is apolar with chains of inverted orientation stacking along *c*, Fig. 7(b). There are no face-to-face π-stacking interactions between the chains. Large channels approximately 11 Å in diameter are created down the *a* axis. TGA indicates a substantial mass loss of *ca.* 35% on heating to 200 °C, which is consistent with loss of the aquo and DMSO ligands and an additional 14 DMSO molecules per formula unit (three of which were located), consistent with the >30% void space calculated from the crystal structure. The crystals were not robust and lose crystallinity on loss of solvent.

### Interpenetrating linked-tubes

The crystalline complex [Cu_2_(L1)_2_(OTf)_2_(NMP)_2_(H_2_O)_2_]·2(OTf)·2NMP **4** was isolated from a mixture of Cu(OTf)_2_ and L1 in NMP where OTf is triflate, CF_3_SO_3_^–^. The crystal structure of **4** was determined in space group *P*1. The given molecular formula represents the asymmetric unit of the structure, although one OTf^–^ was not located, see Fig. S3 ESI.† There are two types of Cu^II^ centre, both with distorted octahedral geometries. Cu1 is coordinated by two *trans* aquo ligands along the Jahn–Teller elongated axis (Cu–O distances 2.392(5) and 2.432(5) Å) and four pyridyl groups of L1 ligands (Cu–N distances 2.013(4) to 2.026(4) Å). Cu2 is coordinated by two *trans* pyridyl groups of L1 ligands (Cu–N distances 1.986(4), 2.008(5) Å), two *trans* NMP ligands (Cu–O distances 1.983(4), 1.991(5) Å) and two *trans* triflate anions on the elongated axis (Cu–O distances 2.321(6), 2.342(5) Å). There are two crystallographically distinct L1 ligands which are enantiomers, and one of which has a solvent NMP as an intra-cavity guest molecule. Both L1 ligands bridge between three Cu^II^ centres.

**Fig. 8:**
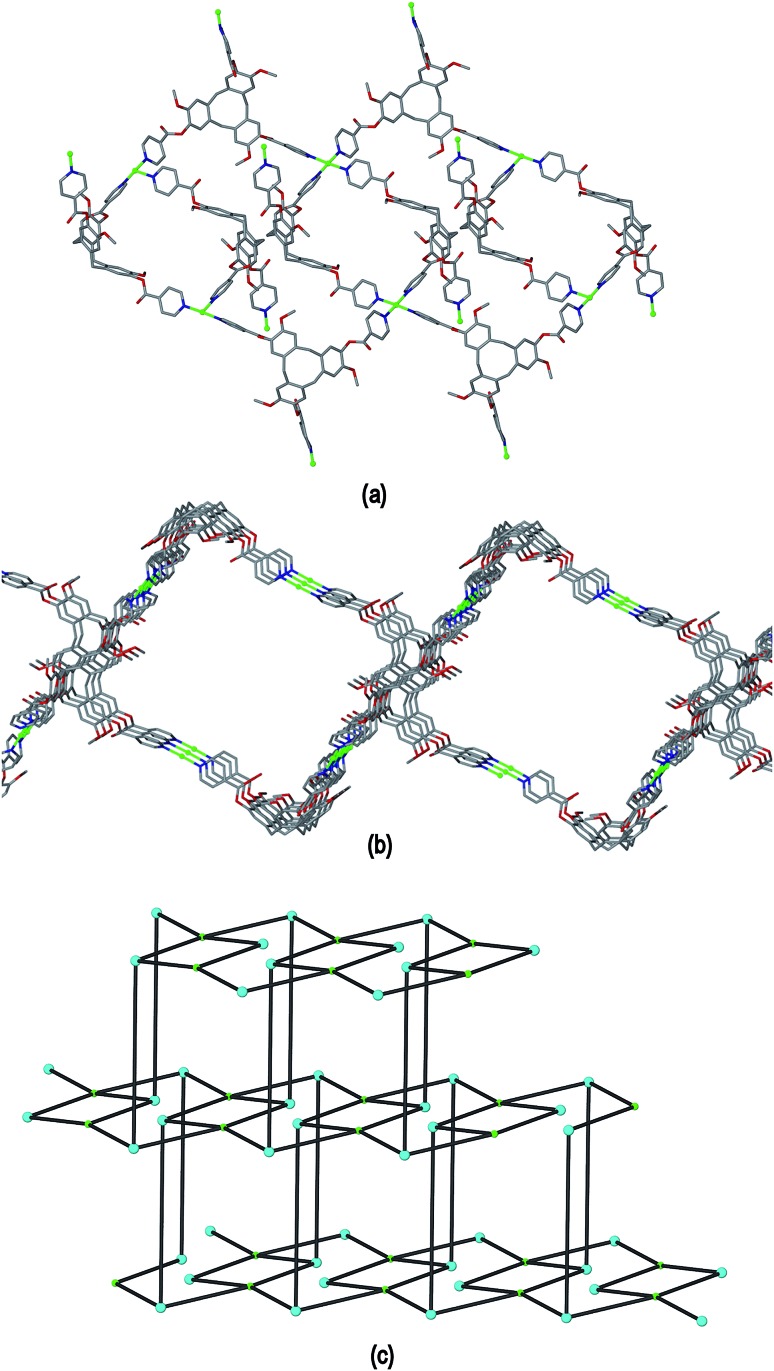
From the crystal structure of [Cu_2_(L1)_2_(OTf)_2_(NMP)_2_(H_2_O)_2_]·2(OTf)·2NMP **4**. (a) Formation of a ladder motif through the 4-connecting Cu2 centres; (b) 2D coordination polymer of linked tubes; (c) connectivity diagram with Cu centres in green and L1 ligand centres larger spheres in blue.

Complex **4** features a cationic [Cu_2_(L1)_2_(OTf)_2_(NMP)_2_(H_2_O)_2_]^2+^ 3,4-connected 2D coordination polymer, noting that Cu2 is topologically trivial in terms of network connectivity and the connecting nodes are the tripodal L1 ligands and the 4-connecting Cu1 which act as a square planar node. The Cu1 centres are linked by L1 ligands into a ladder arrangement shown in Fig. 8(a), these are then linked together above and below their plane through Cu2 centres to form a 2D network that features an series of linked tubular pores of approximate cross-section of 3 nm across the diagonal, Fig. 8(b). The tubes run along the *b* axis. Each connecting centre in the framework is involved in 4-, 6- and 8-membered rings to give a simple framework topology of (4.6^2^.8)(6^2^.8)(4.6^2^.8^2^) noting that there are two types of 3-connecting centre, one in the centre of the ladder motif and the other at the sides, Fig. 8(c). While 2D networks of 3,4-connectivity are known, we are not aware of another example with this topology. Our previously reported examples of 3,4-connected 2D networks with CTV-type ligands had (4^2^.6^2^)(4.6^2^)_2_ topology which forms a sheet rather than tubular motif and was found in complexes of the ligands tris[4-(4-pyridyl)benzoyl]-cyclotriguaiacylene or tris[3-(4-pyridyl)benzoyl]-cyclotriguaiacylene.^12^

**Fig. 9:**
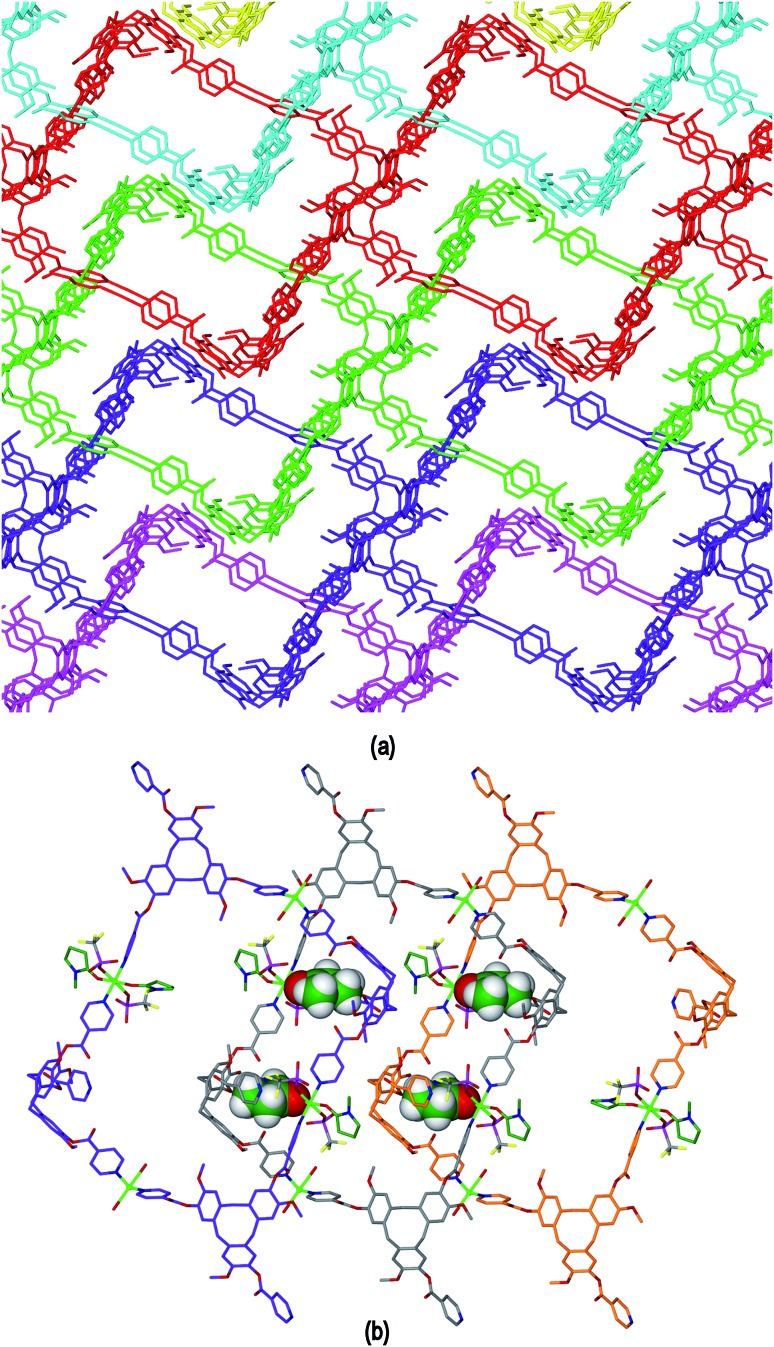
Extended structure of complex **4** showing (a) 2D → 3D network interpenetration with terminal ligands excluded for clarity; (b) detail of three networks illustrating the Cu1–NMP∩L1 host–guest interactions that occur between the networks, guest NMP ligands are shown in space filling mode.

The framework structure of **4** is reminiscent of the tubular 1D coordination polymer carboxylate-appended CTV-type ligands reported by Zheng,^9^ in both examples the tubes are bounded by four CTV scaffolds with molecular bowls directed inwards and linked by Cu^II^ cations. Unlike in Zheng's example, in complex **4** the size of the channels is restricted as the material exhibits 2D → 3D parallel interpenetration, Fig. 9(a). Interpenetration occurs such that each tubular pore of one 2D framework is occupied by sections of two interpenetrating frameworks. There are host–guest interactions between the interpenetrating networks. These occur between one of the NMP ligands attached to Cu1 of one framework and an L1 ligand (of the type shown without a guest NMP in Fig. S3†) of an adjacent framework, and *vice versa*, shown in Fig. 9(b) with the guest NMP ligands in space-filling mode to highlight them. Similar host–guest motifs involving terminal ligands as the guest have been observed in other CTV-type assemblies,^12,20^ and between calixarene-based chains.^6^ Channels are evident along the *a* and *c* axes of the lattice, see ESI Fig. S4.†

### Coordination polymers with common topologies

The coordinating anion cyanide plays a distinct structural role in complex **1**, hence complexes with weakly coordinating anions would be anticipated to form completely different types of network structures. This is indeed the case, and reaction of ligand L1 with [Cu^I^(NCMe)_4_]·BF_4_ in acetonitrile results in isolation of single crystals of complex [Cu(L1)(NCMe)]·BF_4_·1.5(CH_3_CN)·2H_2_O **5**, while similar reaction of L1 with Cu^II^(BF_4_)_2_ in *N*-methylpyrrolidone (NMP) gives complex [Cu_2_(L1)_2_(NMP)(H_2_O)]·4BF_4_·12NMP·1.5H_2_O **6**.

The crystal structure of complex **5** was determined in space group *Pbca* from data collected with synchrotron radiation. The asymmetric unit is composed of one Cu^I^, one L1 and an acetonitrile ligand, a disordered BF_4_^–^, and solvent CH_3_CN and water sites. The Cu^I^ has approximate tetrahedral coordination and is coordinated by one acetonitrile and three symmetry related L1 ligands with Cu–N distances between 2.005(5) and 2.049(5) Å. The L1 ligand has approximate molecular *C*_3_ symmetry and each pyridyl group coordinates, bridging between three symmetry-related Cu^I^ centres.

**Fig. 10:**
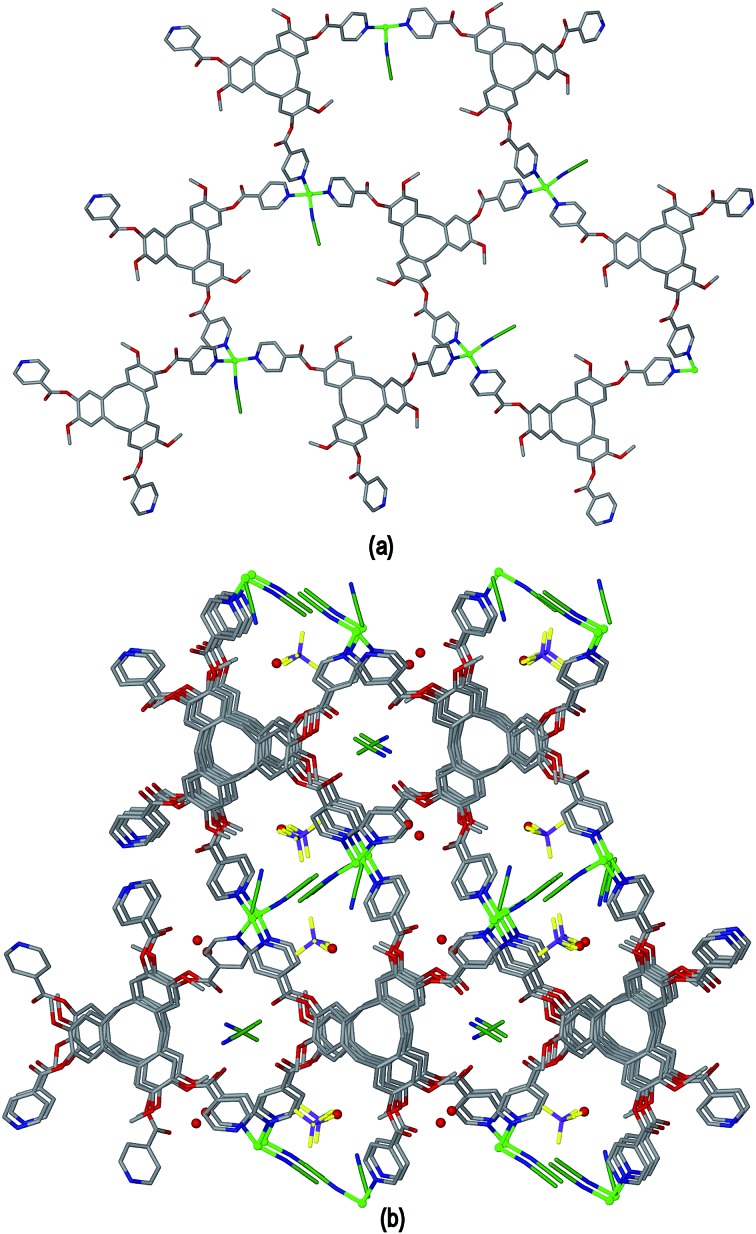
Crystal structure of [Cu(L1)(NCMe)]·BF_4_·1.5(CH_3_CN)·2H_2_O **5**. (a) 2D Coordination polymer network with 6^3^ topology; (b) packing diagram viewed down the *a* axis. Carbon atoms of CH_3_CN shown in dark green and H atoms excluded.

The [Cu(L1)(NCMe)]^+^ complex of **5** is a 2D coordination polymer with a hexagonal network of 6^3^ topology also referred to as hex which forms in the *bc* plane of the crystal lattice, where both the Cu^I^ and L1 components act as 3-connecting centres, Fig. 10(a). The Cu^I^ positions are roughly coplanar but the network shows considerable puckering due to the bowl-shape of L1. Whether the L1 molecular cavities are oriented up or down alternates throughout the network, and the network is racemic, with the L1 enantiomer alternating along the *c* axis. The 2D networks pack along the *a* axis such that there is bowl-in-bowl stacking of the L1 molecular bowls of adjacent coordination polymers throughout the lattice, Fig. 10(b). The ligand enantiomer alternates within each bowl-in-bowl stack, and the stacking L1 ligands have aromatic ring centroid separations *ca.* 4.7 Å, indicating that there are no π–π stacking interactions between them. The *a* unit cell length of 9.459(2) Å is indicative of this type of bowl-in-bowl stacking of CTV-type scaffolds, akin to that observed in α-phase CTV.^30^ In the previously reported example of a coordination polymer involving L1 with a tetrahedral metal centre, namely complex [Ag(L1)_2_][Co(C_2_B_9_H_11_)_2_], a doubly-bridged chain structure is formed and a head-to-head motif is observed between L1 ligands of adjacent chains.^15^ In the overall crystal lattice of 5 channels are formed which are occupied by ligated and solvent acetonitrile, water and disordered BF_4_^–^ counter-anions.

**Fig. 11:**
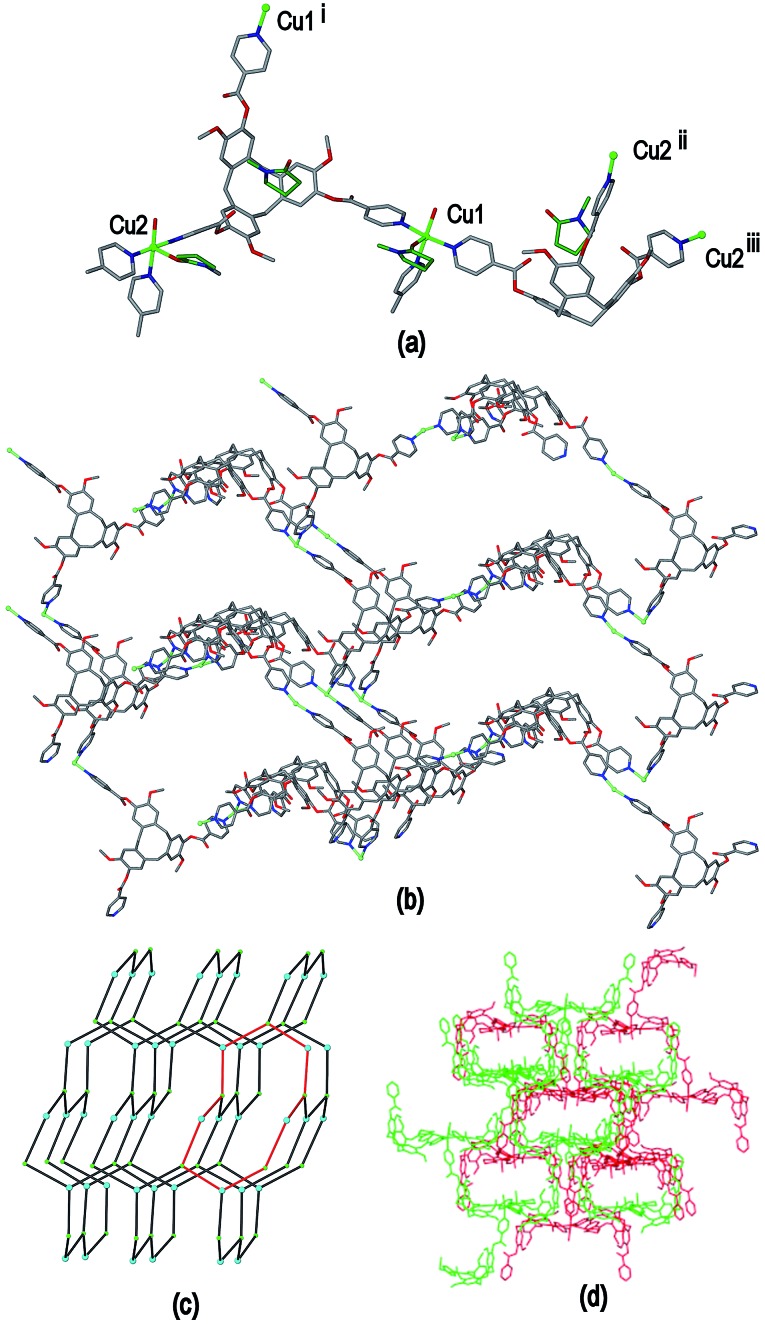
From the crystal structure of [Cu_2_(L1)_2_(NMP)(H_2_O)]·4BF_4_·12NMP·1.5H_2_O **6**. (a) Coordination environment, two types of L1 ligands and host–guest associations between L1 and NMP. For the sake of clarity, only the pyridyl groups of some L1 ligands within the Cu coordination sphere are shown, carbon atoms of NMP in dark green; (b) single [Cu_2_(L1)_2_]^4+^ coordination polymer with terminal ligands excluded; (c) connectivity diagram showing ths or (10,3)-*b* topology with one chair-conformation 10-ring in red, L1 connecting centre is taken as at centre of Cu_3_ plane as this allows for easier identification of network type; (d) two-fold interpenetration of inverted ths nets. Symmetry operations: (i) *x*, 1/2 – *y*, 1/2 + *z*; (ii) *x* – 1, 3/2 – *y*, *z* – 1/2; (iii) *x* – 1, *y*, *z* – 1.

The crystal structure of complex [Cu_2_(L1)_2_(NMP)(H_2_O)]·4BF_4_·12NMP·1.5H_2_O **6** was determined in space group *P*2_1_/*c* and the given formula represents the asymmetric unit, and the cationic [Cu_2_(L1)_2_(NMP)(H_2_O)]^4+^ complex forms a 3D coordination polymer. Both Cu^II^ centres have similar geometries, each being coordinated by pyridyl groups from three L1 ligands, one aquo and one NMP ligand in a distorted square pyramidal fashion, Fig. 11(a). For both Cu^II^ centres the NMP ligand occupies the apical position and Cu–N distances range from 1.985(7) to 2.036(5) Å, Cu–OH_2_ distances 1.959(6) and 2.003(4) Å and apical Cu–O distances 2.169(7) and 2.282(5) Å. If the terminal aquo and NMP ligands are disregarded then the Cu^II^ cations have near T-shaped connectivity, with *trans* angles 159.5(3)° around Cu1 and 178.8(2)° around Cu2, and *cis* N–Cu–N angles between 87.0(2) and 90.4(2)°. Both crystallographically distinct ligand L1 types bridge between three Cu centres, however they are not structurally similar. One has all ester groups arranged with carbonyls pointed towards the centre of the molecular bowl, while the other has only one ester directed inwards. Both contain a guest NMP molecule within their molecular bowls, Fig. 11(a).

Both the Cu centres and the L1 ligands of complex **6** act as 3-connecting centres to form a 3D coordination polymer, Fig. 11(b). The connectivity diagram is shown in Fig. 11(c) and illustrates that the network features 10-membered rings and has the (10,3)-*b* topology which is also referred to as ths topology and is related to the structure of ThSi_2_. This network characterized by zig-zag motifs and chair-conformation 10-membered rings.^31^ Throughout the structure of complex **3** there are two interpenetrating ths networks which are related to one another by inversion, Fig. 11(d). Any channels that run through the interpenetrated network pair are relatively small and are filled with additional NMP solvent and counter-anions (see ESI†). The (10,3)-*b* topology occurs less commonly than other 3-connected networks but a number of examples are known for different coordination polymers.^32^ This is the first report, however, that involves a CTV-type ligand as one of the 3-connecting centres for a (10,3)-*b* net.

**Fig. 12:**
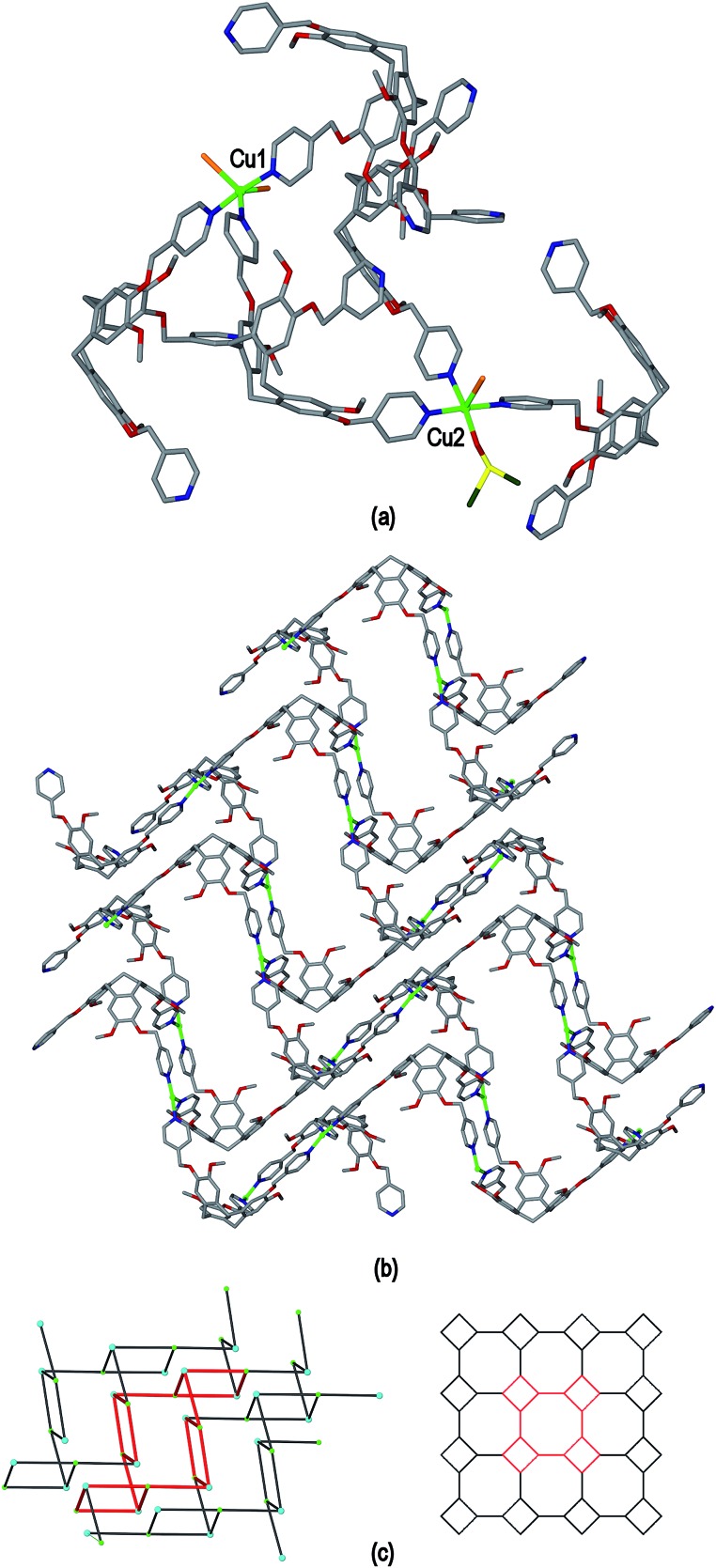
From the crystal structure of [Cu_2_(L2)_2_Br_3_(DMSO)]·Br·3.5DMSO **7**, only one disorder position is shown for disordered L2 moieties. (a) Complete Cu^II^ coordination spheres; (b) 2D [Cu_2_(L2)_2_Br_3_(DMSO)]^+^ coordination polymer with terminal ligands excluded; (c) connectivity diagram (on left, green spheres = Cu^II^; blue spheres = L2 centroid) and idealized 4.8^2^ topology shown on right, in both cases one 8-ring and four 4-rings are highlighted in red.

The use of a coordinating anion with L2 results in the formation of a very different type of 2D coordination polymer structure in the material [Cu_2_(L2)_2_Br_3_(DMSO)]·Br·3.5DMSO **7**. Complex **7** was obtained from vapour diffusion of ethyl acetate into a DMSO solution containing equimolar amounts of L2 and CuBr_2_. The crystal structure was solved in space group *P*1 and the given formula represents the asymmetric unit although an uncoordinated Br^–^ counter-anion and additional solvent molecules were not located in the structure. The two L2 ligands within the asymmetric unit are of different ligand enantiomers. One methyl pyridyl group and one methyl group were both modelled as disordered across two positions, see ESI.† There are two crystallographically distinct Cu^II^ centres, shown in Fig. 12(a). Cu1 has trigonal bipyramidal coordination with two equatorial Br^–^ ligands at Cu–Br distances 2.463(1) and 2.572(1) Å, and three pyridyl ligands from three L2 ligands at Cu–N distances ranging from 2.060(11) to 2.107(5) Å. Cu2 is square pyramidal with an axial Br^–^ ligand (Cu–Br 2.814(2) Å), three L2 pyridyl ligands (Cu–N distances from 1.996(7) to 2.089(7) Å), and a coordinated DMSO at Cu–O distance 2.134(7) Å.

If the terminal ligands are disregarded then both Cu^II^ centres have a similar T-shaped geometry of three L2 ligands. Each L2 ligand bridges between three Cu^II^ centres to form a 3-connected 2D network, Fig. 12(b). The network topology is a highly distorted 4.8^2^ network as shown in Fig. 12(c) where both a connectivity diagram of **7** and an idealized 4.8^2^ network are shown. Networks with this topology have been previously observed for CTV-type coordination polymers.^13^ An ideal 4.8^2^ network is formed from trigonal centres, and the combination of T-shaped and orthogonal connecting centres found here leads to the distortions with the 8-membered rings in a chair-conformation. The 2D coordination polymers of **7** are two-tiered rather than planar. Although the lattice of complex **7** does not show substantial channels, cavities are created by both the networks and the manner in which they pack together (see ESI†). Some solvent DMSO positions were located including an intra-cavity guest position, however TGA indicates the level of solvation of complex **7** is higher than could be established by crystallography (see ESI†). This is also consistent with void calculations.

## Experimental

(±)-Tris(iso-nicotinoyl)cyclotriguaiacylene L1,^15^ (±)-tris(4-pyridylmethyl)cyclotriguaiacylene L2 (ref. 16) and (±)-tris{4-(4-pyridyl)benzyl}cyclotriguaiacylene L3,^17^ were synthesized according to literature methods. All chemicals were obtained from commercial sources and were used without further purification. Infra-red spectra were recorded as solid phase samples on a Perkin-Elmer Spectrometer. Elemental analyses were performed on material that had been washed with diethyl ether, subsequently dried at 80–90 °C under vacuum and then exposed to the atmosphere, hence may show different levels of solvation to those established by crystallography or TGA due to solvent loss and/or absorption of atmospheric water.

### [Cu^I^_4_Cu^II^_1.5_(L1)_3_(CN)_6_]·CN·*n*(DMF) **1**

L1 (10 mg, 0.014 mmol) and CuCN (5 mg) were dissolved in DMF with gentle heating. The solution was left to stand for three weeks and orange hexagonal crystals formed which were suitable for X-ray crystallography. IR (solid state): *ν*(cm^–1^) 2127 (Cu–*CN*), 1743 (CO_2_R), 1706, 1652, 1613 (C=C), 1506, 1412, 1324, 1261, 1204, 1175, 1137, 1099, 1059, 1003, 849, 750. Cu_5.5_(C_42_H_33_N_3_O_9_)_3_(CN)_7_(C_3_H_7_NO)_4_(H_2_O)_10_ requires C 54.85, H 4.67, N 8.82%; found C 54.25, H 3.85, N 8.85%.

### [Cu_3_(L2)_4_(H_2_O)_3_]·6(OTf)·*n*(DMSO) **2**

L2 (10 mg, 0.015 mmol) was dissolved in DMSO and a Cu(OTf)_2_ solution (0.5 ml, 0.02 mmol) in DMSO was added. Crystals were grown by vapour diffusion of diethyl ether into the mixture. Blue/lilac plate-like crystals were obtained which were suitable for X-ray crystallography (2 mg). IR (solid state): *ν*(cm^–1^) 1620 (C=C), 1508, 1447, 1430, 1398, 1254, 1222, 1145, 1087, 1066, 1028, 943, 844, 809, 744, 636. Cu_3_(C_42_H_39_N_3_O_6_)_4_(CF_3_SO_3_)_6_(C_2_H_6_SO)_5_ requires C 52.58, H 4.46, N 4.00%; found C 51.32, H 4.30, N 4.10%.

### Complex **2**@C_60_

As synthesised crystals of **2** were soaked in a saturated toluene solution of C_60_ and left for two weeks. The crystals turned dark brown/red. Cu_3_(C_42_H_39_N_3_O_6_)_4_(CF_3_SO_3_)_6_(C_60_) requires C 62.00, H 3.47, N 3.71%; found C 62.80, H 3.20, N 3.10%.

### [Cu_2_(L3)_2_Br_2_(H_2_O)(DMSO)]·2Br·*n*(DMSO) **3**

A solution of CuBr_2_ (5.4 mg, 0.024 mmol) in DMSO (0.5 ml) was added to a solution of L3 (15 mg, 0.016 mmol) in DMSO (1 ml). Ethyl acetate vapour diffusion into the solution resulted in dark green crystals which were filtered off, washed with diethyl ether and dried *in vacuo* (16 mg). IR (solid state): *ν*(cm^–1^) 3003, 2913, 1613, 1510, 1464, 1434, 1397, 1342, 1313, 1260, 1218, 1193, 1144, 1084, 1022, 949, 878, 852, 816, 758, 738, 710, 666, 615, 566. Found C 60.90, H 4.75, N 3.75, S 2.05; Cu(C_60_H_51_N_3_O_6_)Br_2_(C_2_H_6_SO)(H_2_O) requires C 60.56, H 4.84, N 3.42, S 2.61%; found C 60.90, H 4.75, N 3.75, S 2.05%.

### [Cu_2_(L1)_2_(OTf)_2_(NMP)_2_(H_2_O)_2_]·2(OTf)·2NMP **4**

L1 (10 mg, 0.014 mmol) was dissolved in NMP (1 ml) and Cu(OTf)_2_ (10 mg, 0.028 mmol) in NMP (0.5 ml) was added. Crystals were grown by vapour diffusion using diethyl ether as the antisolvent. Blue plates formed over two weeks which where suitable for X-ray crystallography. IR (solid state): *ν*(cm^–1^) 3383, 2939, 1748 (CO_2_R), 1651 (s), 1564, 1507, 1447, 1421, 1404, 1323, 1260 (s, w), 1223, 1177, 1140, 1104, 1059, 1029, 1004, 929, 857, 753, 697, 679, 635. Cu(C_42_H_33_N_3_O_9_)(CF_3_SO_3_)_2_(C_5_H_9_NO)_4_(H_2_O)_3_ requires C 50.05, H 4.90, N 6.38%, found C 50.10, H 4.90, N 6.70%.

### [Cu(L1)(NCMe)]·BF_4_·2(CH_3_CN)·H_2_O **5**

L1 (10 mg, 0.014 mmol) was dissolved in NMP (1 ml) and Cu(MeCN)_4_BF_4_ (5 mg, 0.016 mmol) was added. The yellow solution was set up for vapour diffusion with diethyl ether as an antisolvent. Yellow crystals were grown over two weeks which were suitable for X-ray crystallography. IR (solid state): *ν*(cm^–1^) 1743 (CO_2_R), 1611 (C=C), 1507, 1416, 1325, 1264, 1206, 1175, 1136, 1111, 1056 (B–F), 852 (B–F), 757 (B–F). Both microanalysis and powder XRD indicate that the bulk sample is not pure. Cu(C_42_H_33_N_3_O_9_)(BF_4_)(MeCN)_3_(H2O) requires C 56.79, H 4.37, N 8.28%; found C 46.05, H 3.90, N 10.05%.

### [Cu_2_(L1)_2_(NMP)(H_2_O)]·4BF_4_·12NMP·1.5H_2_O **6**

L1 (10 mg, 0.014 mmol) was dissolved in nitromethane (1 ml) and a Cu(BF_4_)_2_ solution (0.5 ml, 0.02 mol) in NMP was added. Crystals were grown by vapour diffusion using diethyl ether as the antisolvent. Blue plates formed over three weeks which where suitable for X-ray crystallography. IR (solid state): *ν*(cm^–1^) 1747 (CO_2_R), 1611 (C=C), 1507, 1477, 1444, 1416, 1400, 1325, 1264, 1206, 1175, 1136, 1111, 1056 (B–F), 1034, 1005, 941, 911, 852 (B–F), 757 (B–F), 725. Cu(C_42_H_33_N_3_O_9_)(BF_4_)_2_(C_5_H_9_NO)(H_2_O)_2_ requires C 51.50, H 4.23, N 5.11%; found C 51.30, H 4.60, N 5.50%.

### [Cu_2_(L2)_2_Br_3_(DMSO)]·Br·*n*(DMSO) **7**

A solution of CuBr_2_ (20 mg, 0.090 mmol) in DMSO (2 ml) was added to a solution of L2 (60 mg, 0.088 mmol) in DMSO (5 ml). Ethyl acetate vapour diffusion into the solution resulted in dark green crystals of which were filtered off, washed with ethyl acetate and dried *in vacuo*. Yield: 72 mg. IR (solid state): *ν*(cm^–1^) 1619, 1607, 1508, 1481, 1445, 1427, 1397, 1344, 1263, 1218, 1192, 1147, 1187, 1025, 948, 845, 810, 747, 701, 617, 492. Found C 52.95, H 4.50, N 4.00, S 2.60%; Cu(C_42_H_39_N_3_O_6_)Br_2_(C_2_H_6_SO) requires C 53.75, H 4.61, N 4.27, S 3.25%.

### X-ray crystallography

X-ray diffraction data were collected at low temperatures with Cu-*Kα* radiation (*λ* = 1.54184 Å) (complexes **1**, **2**, **4**, **6**), Mo-*Kα* radiation (*λ* = 0.71073 Å) (complexes **3**, **7**), or using synchrotron radiation (*λ* = 0.6889 Å) (complex **5**). Data were corrected for absorption using a multi-scan method, and structures were solved by direct methods using SHELXS-97 and refined by full-matrix or block-matrix (complex **6**) least squares on *F* ^2^ by SHELXL-97.^33^ For all complexes aside from **5** and **6** the structures contained significant void space and residual electron density that could not be meaningfully refined as additional solvent or counter-anions, hence the SQUEEZE^34^ routine of PLATON^35^ was employed. In general the crystals were weakly diffracting as is commonly the case for coordination polymer materials where a low percentage of the unit cell volume is occupied by the ordered framework.

#### Complex **1**

C_133_H_99_Cu_5.5_N_17_O_31_, hexagonal *a* = *b* = 29.5994(13), *c* = 52.255(4) Å, space group *R*3, *θ*_max_ = 51.44°, *λ* = 1.54184 Å, data/restraints/parameters 9343/0/424, *R*_1_ = 0.0881, w*R*_2_ = 0.2828.

#### Complex **2**

C_174_H_154_Cu_3_F_18_N_12_O_31_S_6_, monoclinic *a* = 57.029(2), *b* = 34.131(3), *c* = 24.4704(6) Å, *β* = 95.496(2)°, space group *C*2/*c*, *θ*_max_ = 44.49°, *λ* = 1.54184 Å, data/restraints/parameters 18 507/0/953, *R*_1_ = 0.0900, w*R*_2_ = 0.2508.

#### Complex **3**

C_128_H_126_Br_4_Cu_2_N_6_O_17_S_4_, monoclinic *a* = 11.0753(10), *b* = 32.119(3), *c* = 47.079(4) Å, *β* = 92.659(1)°, space group *P*2_1_/*n*, *θ*_max_ = 20.00°, *λ* = 0.71073 Å, data/restraints/parameters 77 638/396/1300, *R*_1_ = 0.1460, w*R*_2_ = 0.4168.

#### Complex **4**

C_108_H_102_Cu_2_F_12_N_10_O_36_S_4_, triclinic *a* = 14.4856(3), *b* = 18.0721(4), *c* = 30.4166(7) Å, *α* = 96.787(2), *β* = 93.193(2), *γ* = 102.088(2)°, space group *P*1, *θ*_max_ = 62.21°, *λ* = 1.54184 Å, data/restraints/parameters 23 197/2/1452, *R*_1_ = 0.1063, w*R*_2_ = 0.3235.

#### Complex **5**

C_47_H_44.5_BCuF_4_N_5.5_O_11_, orthorhombic *a* = 9.459(2), *b* = 29.785(7), *c* = 34.465(8) Å, space group *Pbca*, *θ*_max_ = 25.0°, *λ* = 0.6889 Å, data/restraints/parameters 9346/2/640, *R*_1_ = 0.1163, w*R*_2_ = 0.3652.

#### Complex **6**

C_149_H_187_B_4_Cu_2_F_16_N_19_O_34.5_, monoclinic *a* = 28.9123(10), *b* = 20.7706(8), *c* = 29.7284(13) Å, *β* = 101.224(4)°, space group *P*2_1_/*c*, *θ*_max_ = 60.00°, *λ* = 1.54184 Å, data/restraints/parameters 27 015/23/2026, *R*_1_ = 0.1262, w*R*_2_ = 0.3928.

#### Complex **7**

C_93_H_105_Br_4_Cu_2_N_6_O_16.5_S_4.5_, triclinic *a* = 15.4223(10), *b* = 19.5085(12), *c* = 28.1607(17)(15) Å, *α* = 100.656(4), *β* = 105.671(3), *γ* = 102.750(4)°, space group *P*1, *θ*_max_ = 25.00°, *λ* = 0.71073 Å, data/restraints/parameters 27 015/47/1044, *R*_1_ = 0.1149, w*R*_2_ = 0.3488.

Further details of data collections and structure refinements are given in the ESI.†

## Conclusions

Using molecular hosts as ligands to engineer different types of cavity spaces within metal–organic frameworks requires both that the molecular host acts as a bridging ligand and that the molecular cavity is potentially accessible to new guest molecules. The coordination polymers reported here and elsewhere^9–15^ demonstrate that tris-ligand-functionalised CTV-type molecular hosts are excellent tectons for the self-assembly of metal–organic frameworks. The ability to predict the framework topologies that are produced remains elusive however, and both common and unusual topologies may result. In the examples reported here seven quite different frameworks are produced according to metal oxidation state and hence geometric preferences, the nature of the counter-anion and whether or not it coordinates, and relatively small differences in the nature of the ligand. L1 and L2 for example differ only in the former having a carbonyl compared with methylene group in the latter yet their respective assemblies with Cu(OTf)_2_ are markedly different – L1 giving a twofold interpenetrating 3-connecting network of ths (10,3)-*b* topology in NMP while L2 gives a highly unusual 2D network structure of linked metallo-cryptophane units of (4^3^.12^3^)(4^2^.12^2^) topology from DMSO, which like NMP is a coordinating solvent. Likewise L1 and CuBr_2_ give a previously reported Borromean chainmail arrangement of Cu_6_L_6_ metallacycles,^20^ while L2 and CuBr_2_ has a 2D coordination polymer structure of 4.8^2^ topology.

Host–guest relationships may form within or between networks that render embedded molecular recognition site of the ligands inaccessible, and this is illustrated here by complex **5** where bowl-in-bowl stacking occurs between L1 ligands of adjacent coordination polymers, and to a lesser extent in complex **4** where half the L1 ligands of a network play host to terminal ligand groups of an adjacent network. The rigid pyramidal shape of the tribenzo[*a*,*d*,*g*]cyclononatriene core of ligands L1–L3 mean that the coordinating 4-pyridyl groups are often orthogonal to one another and this leads to 2D and chain networks which are significantly distorted from planarity and leads to formation of tubular like arrays such as in complex **4** or promotes formation of head-to-head linking of the ligands to form cage (complex **1**) and capsule metallo-cryptophane (complexes **2** and **3**) motifs within the framework. The cage and capsule-embedded frameworks in particular have molecular guest binding sites that are potentially available to new guest molecules, along with large channels throughout the ordered crystal lattice. Although these materials do not withstand complete evacuation of mother liquor, they exhibit new and highly promising structural types for the formation of porous materials with hierarchical pore spaces, and point to an important design principle – the networking of cage or capsule motifs.
