# Supplementary material for: Copper coordination polymers from cavitand ligands: hierarchical spaces from cage and capsule motifs, and other topologies
Source: Chem Sci. 2015 Jul 14;6(10):5779–92. doi: 10.1039/c5sc01801c (PMC5520774; doi:10.1039/c5sc01801c)
Supplement: Supplementary file 1 [file SC-006-C5SC01801C-s001.pdf]

Electronic Supplementary Material (ESI) for Chemical Science.
This journal is © The Royal Society of Chemistry 2015

Copper coordination polymers from cavitand ligands: hierarchical spaces from cage and capsule motifs, and other topologies.

*Flora L. Thorp-Greenwood, Tanya K. Ronson, Michaele J. Hardie**

Supplementary Material

Further details of X-Ray Crystallography

Crystals were mounted under oil on a MiTeGen tip or glass fiber and X-ray diffraction data collected at 100(1), 120(1) or 150(1) *K* with Cu-$K\alpha$ radiation (λ = 1.54184 Å) with an Agilent SuperNova diffractometer (complexes **1**, **2**, **4**, **6**), Mo-$K\alpha$ radiation (λ = 0.71073 Å) using a Bruker Nonius X-8 diffractometer with FR591 rotating anode generator (complexes **3**, **7**), or using synchrotron radiation (λ = 0.6889 Å) with a Rigaku Saturn at station I19 at the Diamond Light Source (complex **5**). Data sets were corrected for absorption using a multi-scan method, and structures were solved by direct methods using SHELXS-97 and refined by full-matrix or block-matrix (complex **6**) least squares on F^2 by SHELXL-97.[1] In general, all non-hydrogen atoms were refined anisotropically and hydrogen atoms were included as invariants at geometrically estimated positions, unless specified otherwise. For all complexes aside from **5** and **6** the structures contained significant void space and residual electron density that could not be meaningfully refined as additional solvent or counter-anions, hence the SQUEEZE [2] routine of PLATON [3] was employed. Details of data collections and structure refinements are given in Table S1, and additional details are given below. In general the crystals were weakly diffracting as is commonly the case for coordination polymer materials with a low percentage of the unit cell volume being occupied by the ordered framework.

Three aromatic rings of complex **1** were refined with rigid body constraints. The structure shows signs of disorder and attempts were made to model this however the poor observed data to parameter ration did not allow for adequate modelling of disorder. Data was also collected for this material using synchrotron radiation but was not of better quality in terms of internal consistency nor overall intensity. For complex **3** DMSO and some disordered lattice Br^- were refined isotropically, and not all Br- disordered sites located. Two aromatic rings were refined with rigid body constraint and the structure was refined with global displacement parameter restraints. One triflate anion of complex **4** was not located in the difference map, and bond length restraints were placed on two C-N bond lengths. One solvent NMP molecule was refined isotropically. The crystals

of complex **5** were of very low quality as evidenced by the high Rint value and requirement for data collection with synchrotron radiation. The BF_4^- anion was modeled with disordered F, and some CH_3CN lattice sites were refined as disordered and at 0.5 occupancy, and a disordered water at 0.7:0.3 occupancies. All water oxygen atoms and disordered CH_3CN were refined isotropically and with FLAT and bond length restraints on one. Bond length restraints were employed for several NMP molecules of complex **6** and some were refined at 0.5 occupancy. One NMP showed significant disordered and hydrogen positions were not calculated for this molecule. Some NMP molecules were refined isotropically and restraints were used on the displacement parameters of one other. Complex **7** the lattice Br^- could not be located in the difference map. Restraints were placed on some displacement parameters, DMSO and disordered groups were refined isotropically, the DMSO ligand was refined with common U_{iso} for the methyl groups and bond length restraints. One pyridyl was refined across two disorder positions each at 0.5 occupancy, and one methyl group refined as disordered at 0.55, 0.45 occupancy.

The structures have been deposited in the Cambridge Crystallographic Data Centre (http://www.ccdc.cam.ac.uk/pages/Home.aspx) deposition numbers CCDC 1401250-1401256.

Table S1: Details of data collections and structure refinements (continued next page)

	**1**[a]	**2**[a]	**3**[b]	**4**[a]
Formula	$C_{132}H_{99}Cu_{5.5}N_{16}O_{31}$	$C_{174}H_{154}Cu_3F_{18}N_{12}O_{45}S_6$	$C_{128}H_{126}Br_4Cu_2N_6O_{17}S_4$	$C_{108}H_{102}Cu_2F_{12}N_{10}O_{36}S_4$
Mr	2780.76	3858.07	2595.31	2599.32
CCDC	1401250	1401251	1401252	1401253
Crystal size (mm)	0.1 x 0.1 x 0.02	0.1 x 0.08 x 0.03	0.20 x 0.10 x 0.05	0.2 x 0.1 x 0.1
Crystal system	Hexagonal	Monoclinic	Monoclinic	Triclinic
Space group	*R*-3	*C*2/*c*	$P2_1/n$	*P*-1
a (Å)	29.5994(13)	57.029(2)	11.0753(10)	14.4856(3)
b (Å)	29.5994(13)	34.131(3)	32.119(3)	18.0721(4)
c (Å)	52.255(4)	24.4704(6)	47.079(4)	30.4166(7)
α (^{0})	90	90	90	96.787(2)
β (^{0})	90	95.496(2)	92.659(1)	93.193(2)
γ (^{0})	120	90	90	102.088(2)
V (Å^3)	39648(4)	47412(5)	16729(3)	7704.9(3)
Z	6	2	4	2
T (K)	100(1)	120(1)	150(1)	120(1)
ρ_{calc} (g.cm^{-3})	0.699	0.541	1.030	1.120
θ range (^{0})	2.99-51.44	3.35-44.49	1.34-20.00	3.08-62.21
No. data collected	23481	41076	77638	52923
No. unique data	9343	18507	15594	23197
R_{int}	0.0540	0.0861	0.0765	0.0294
No. obs. Data	3405	11082	8101	16042

($I > 2\sigma(I)$)				
No. parameters	424	953	1300	1452
No. restraints	0	0	396	2
R_1 (obs data)	0.0881	0.0900	0.1460	0.1063
wR_2 (all data)	0.2828	0.2508	0.4168	0.3235
S	0.774	0.946	1.402	1.225

[a] Cu-$K\alpha$ radiation; [b] Mo-$K\alpha$ radiation; [c] synchrotron radiation

Table S1: (continued) Details of data collections and structure refinements

	**5**[c]	**6**[a]	**7**[b]
Formula	$C_{47}H_{44.5}BCuF_4N_{5.5}O_{11}$	$C_{149}H_{187}B_4Cu_2F_{16}N_{19}O_{34.5}$	$C_{93}H_{105}Br_4Cu_2N_6O_{16.5}S_{4.5}$
Mr	1012.73	3270.50	2161.89
CCDC	1401254	1401255	1401256
Crystal size (mm)	0.20 x0.10 x 0.10	0.1 x 0.1 x 0.05	0.15 x 0.15 x 10.15
Crystal system	Orthorhombic	Monoclinic	Triclinic
Space group	*Pbca*	$P2_1/c$	*P*-1
a (Å)	9.459(2)	28.9123(10)	15.4223(10)
b (Å)	29.785(7)	20.7706(8)	19.5085(12)
c (Å)	34.465(8)	29.7284(13)	28.1607(17)
α (^{0})	90	90	100.656(4)
β (^{0})	90	101.224(4)	105.671(3)
γ (^{0})	90	90	102.750(4)
V (Å^3)	9710(4)	17511.2(12)	7682.0(8)
Z	8	4	2
T (K)	150(1)	120(1)	150(1)
ρ_{calc} (g.cm^{-3})	1.386	1.241	0.9345
θ range (^{0})	1.15-25.00	3.12-60.00	0.78-25.00
No. data collected	66625	49512	154927
No. unique data	9346	25927	27015
R_{int}	0.2239	0.0587	0.0607
No. obs. Data ($I > 2\sigma(I)$)	7124	15144	16957
No. parameters	640	2026	1044
No. restraints	2	23	47
R_1 (obs data)	0.1163	0.1262	0.1149
wR_2 (all data)	0.3652	0.3928	0.3488
S	1.319	1.306	1.249

[a] Cu-$K\alpha$ radiation; [b] Mo-$K\alpha$ radiation; [c] synchrotron radiation

Additional Diagrams for crystal structures

$[Cu^{I}_{4}Cu^{II}_{1.5}(L1)_3(CN)_6]\cdot CN\cdot n(DMF)$ 1.

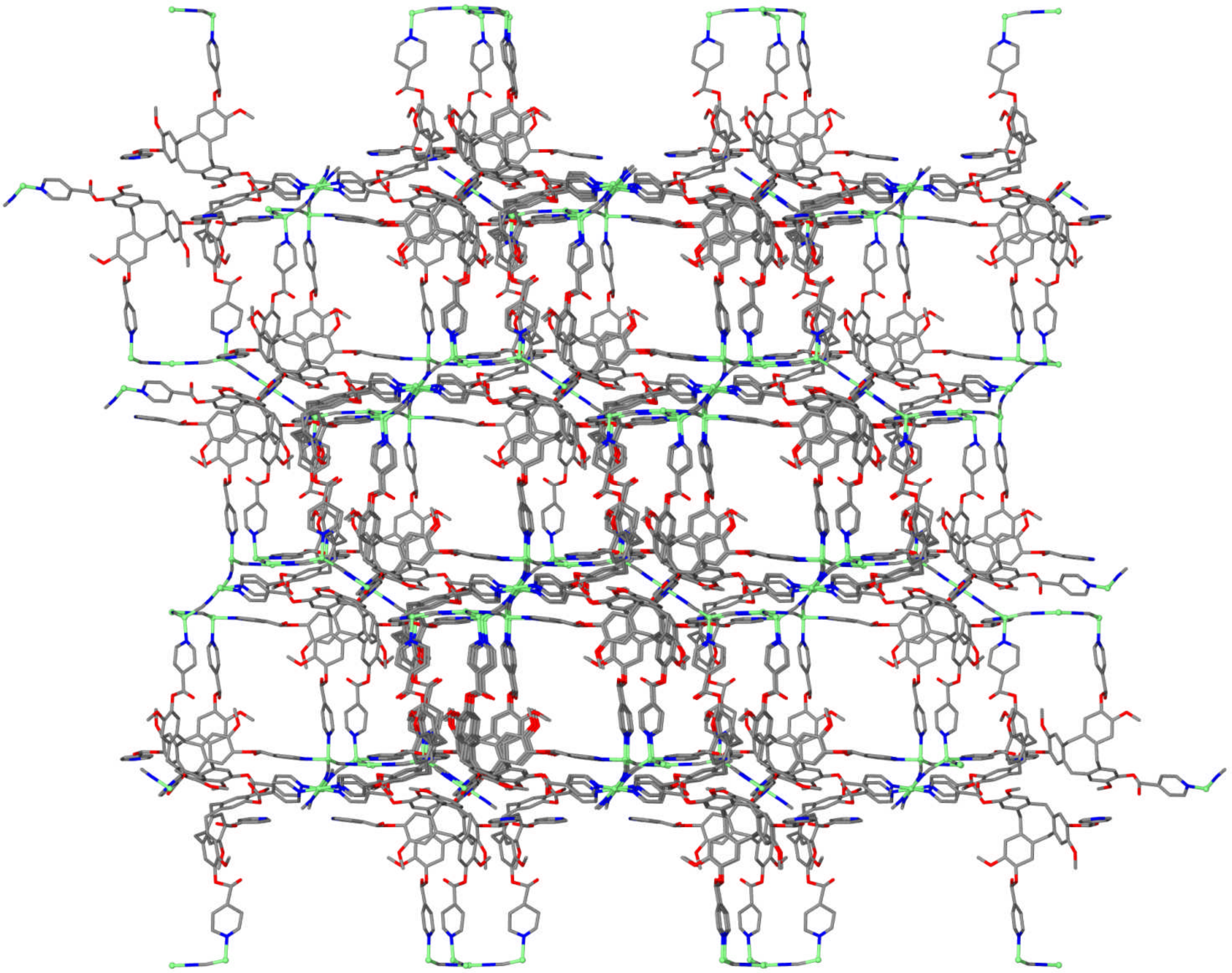

Figure S1: Packing diagram of the crystal structure of complex $[Cu^{I}_{4}Cu^{II}_{1.5}(L1)_3(CN)_6]\cdot CN\cdot n(DMF)$ **1.**

$[Cu_3(L2)_4(H_2O)_3]\cdot6(OTf)\cdot n(DMSO)$ 2

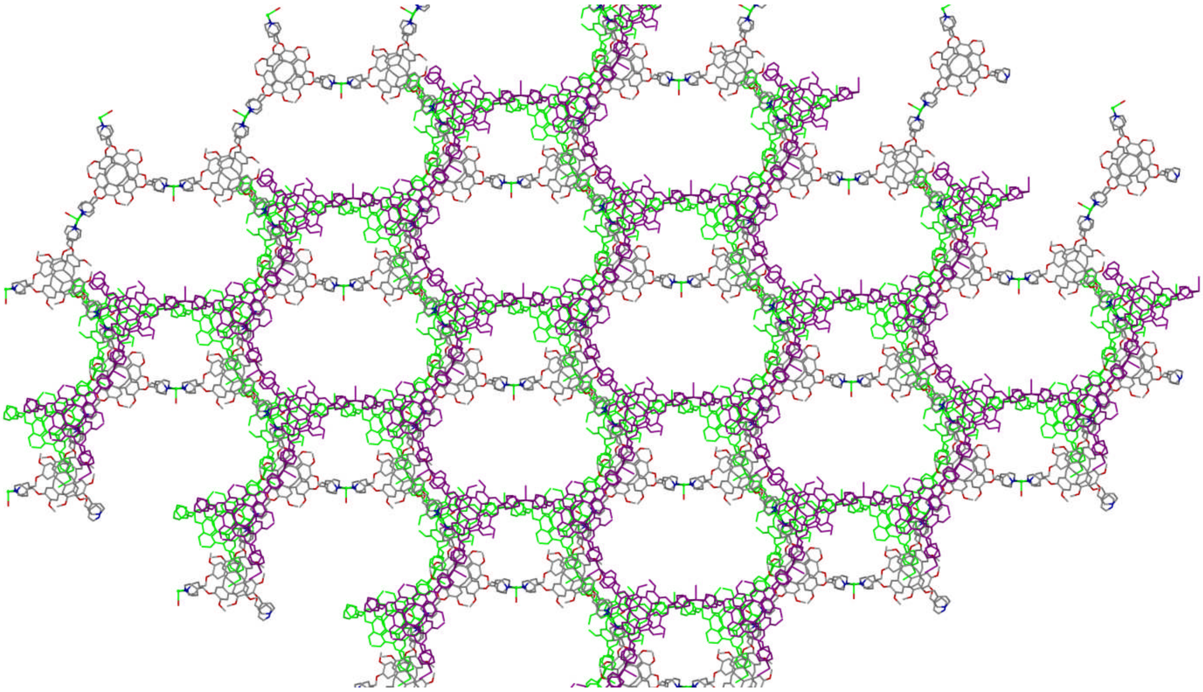

Figure S2: ABA stacking of three 2D coordination polymers within complex $[Cu_3(L2)_4(H_2O)_3]\cdot6(OTf)$ **2.**

$[Cu_2(L1)_2(OTf)_2(NMP)_2(H_2O)_2]\cdot2(OTf)\cdot2NMP$ 4

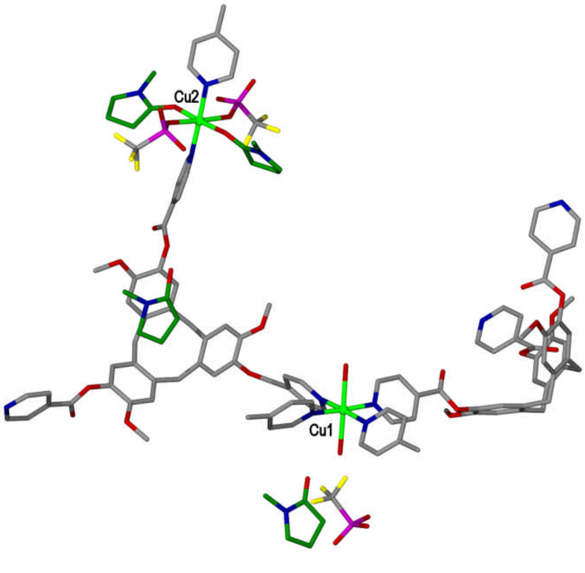

Figure S3. Asymmetric unit and completed Cu^{II} coordination spheres of the crystal structure of $[Cu_2(L1)_2(OTf)_2(NMP)_2(H_2O)_2]\cdot2(OTf)\cdot2NMP$ **4**. Only the pyridyl groups of some L1 ligands within the Cu coordination spheres are shown, carbon atoms of NMP given in dark green.

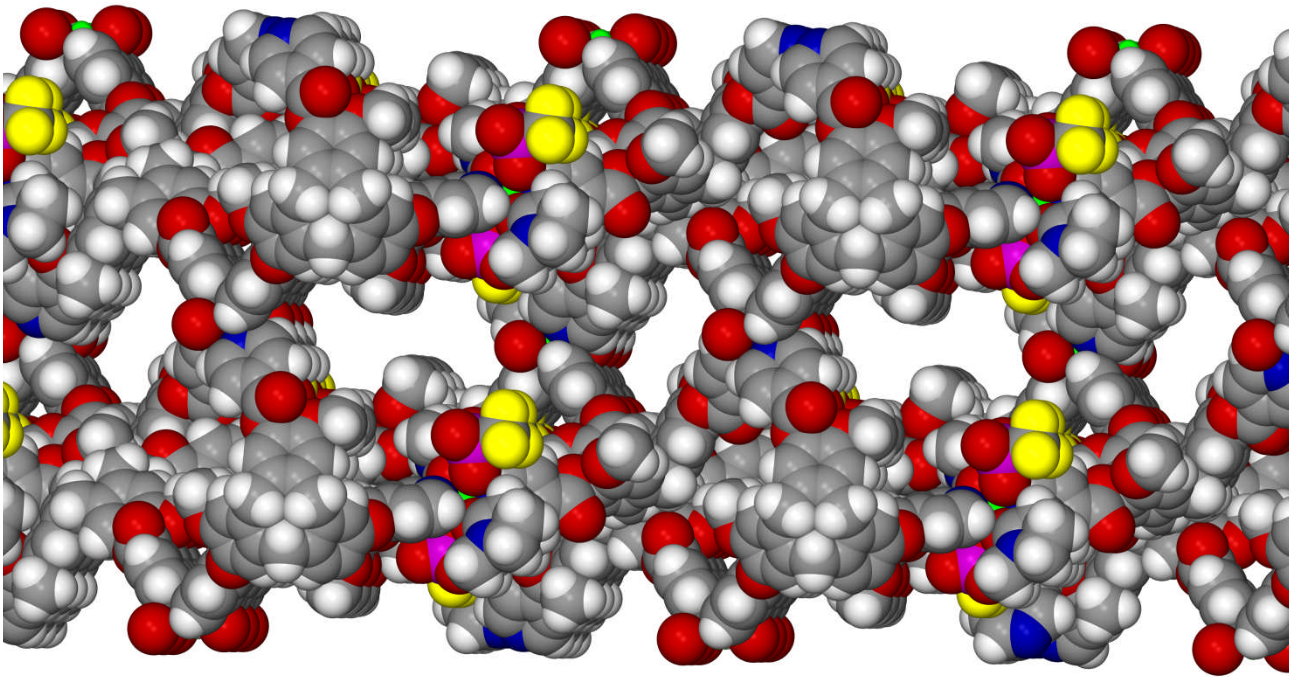

Figure S4: From complex $[Cu_2(L1)_2(OTf)_2(NMP)_2(H_2O)_2]\cdot 2(OTf)\cdot 2NMP$ **4** space-filling packing diagram of the network viewed down *a* with lattice solvent and uncoordinated anions excluded.

$[Cu_2(L1)_2(NMP)(H_2O)]\cdot 4BF_4\cdot 12NMP\cdot 1.5H_2O$ 6.

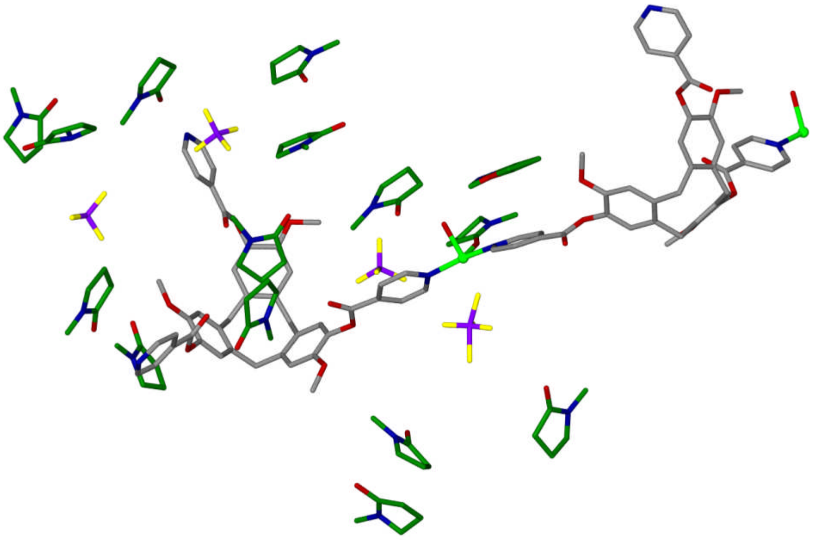

Figure S5. Asymmetric unit of complex $[Cu_2(L1)_2(NMP)(H_2O)]\cdot 4BF_4\cdot 12NMP\cdot 1.5H_2O$ **6**.

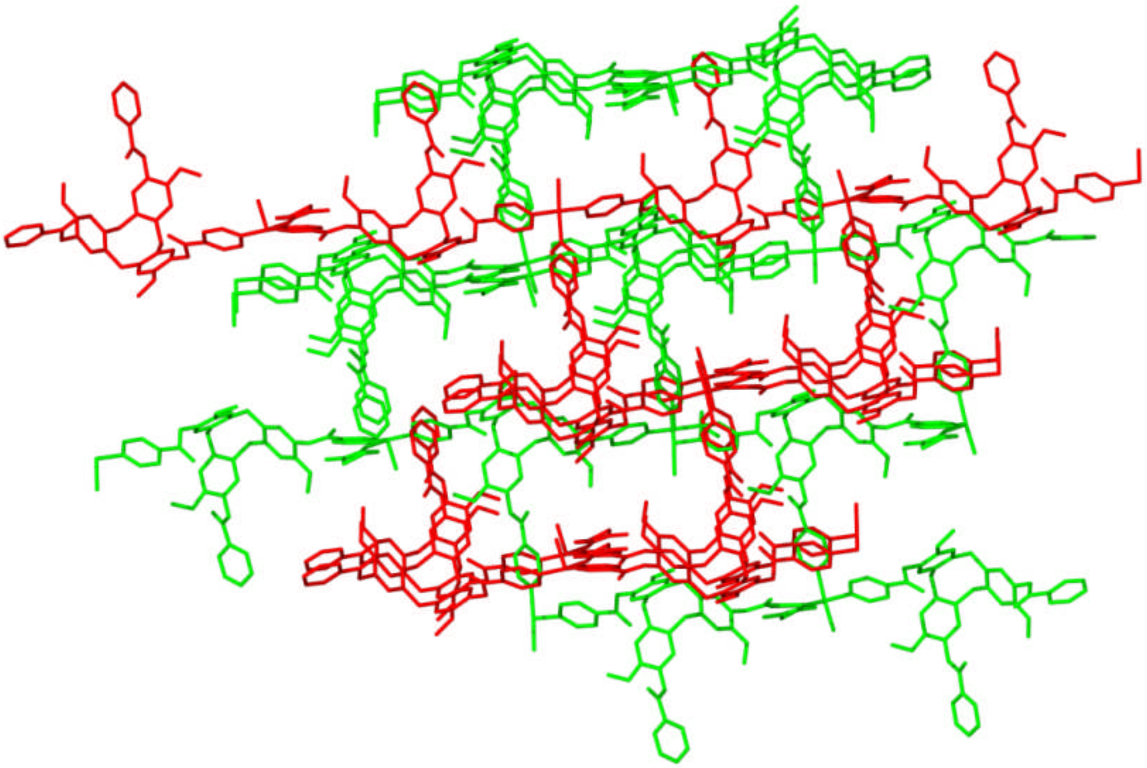

Figure S6. Two interpenetrating $[Cu_2(L1)_2(NMP)(H_2O)]^{4+}$ networks of $[Cu_2(L1)_2(NMP)(H_2O)]\cdot4BF_4\cdot12NMP\cdot1.5H_2O$ **6**.

Infrared Spectroscopy

$[Cu^{I}_{4}Cu^{II}_{1.5}(L1)_3(CN)_6]\cdot CN\cdot n(DMF)$ 1.

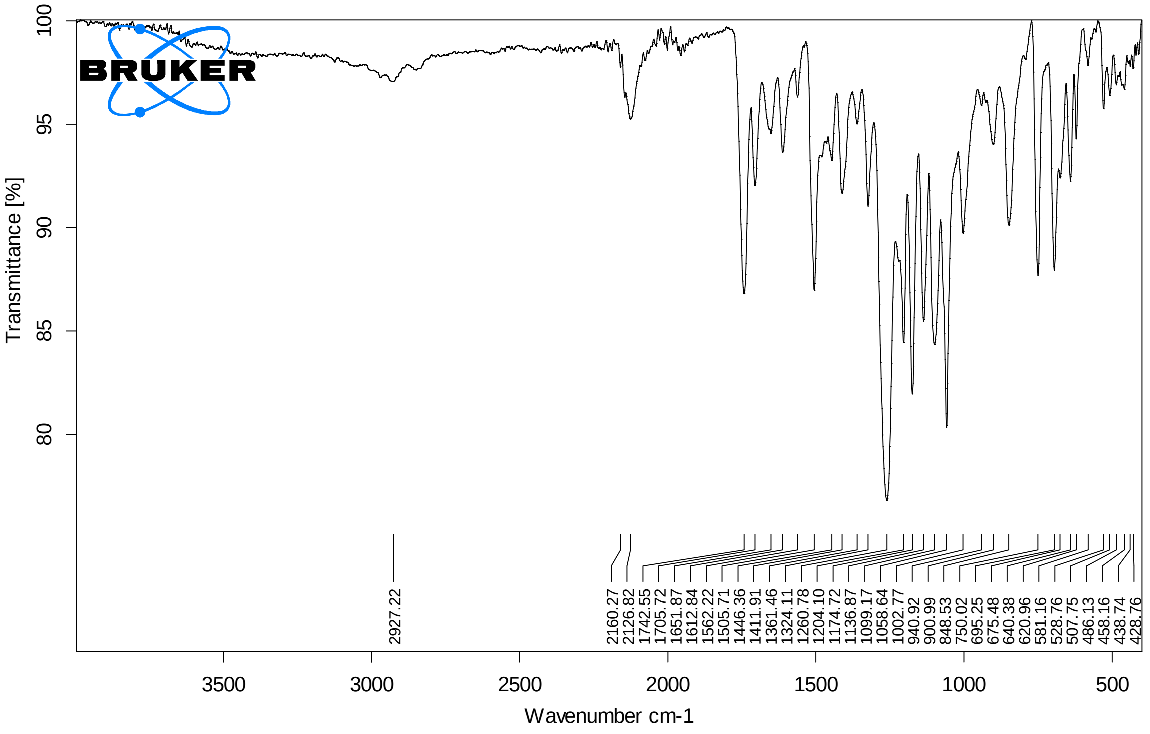

$[Cu_3(L2)_4(H_2O)_3]\cdot 6(OTf)\cdot n(DMSO)$ 2

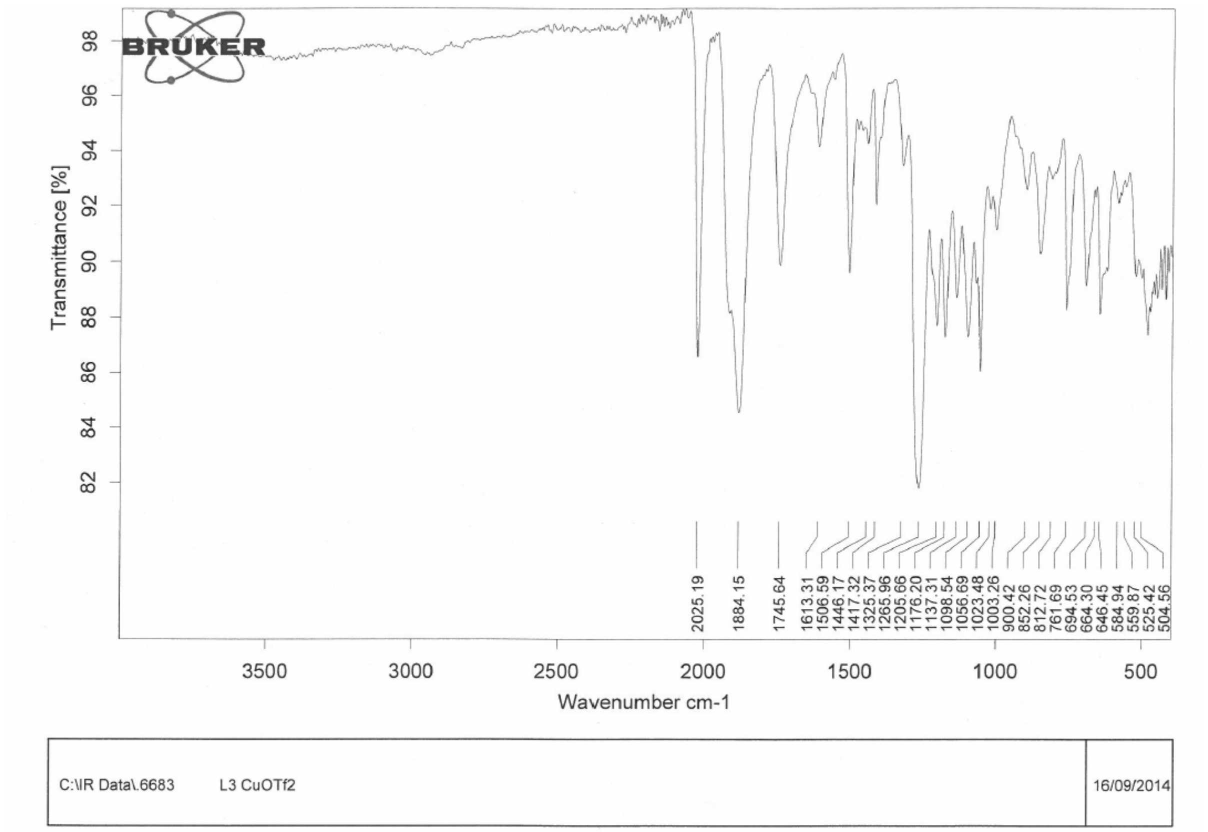

$[Cu_2(L3)_2Br_2(H_2O)(DMSO)]\cdot 2Br\cdot n(DMSO)$ 3

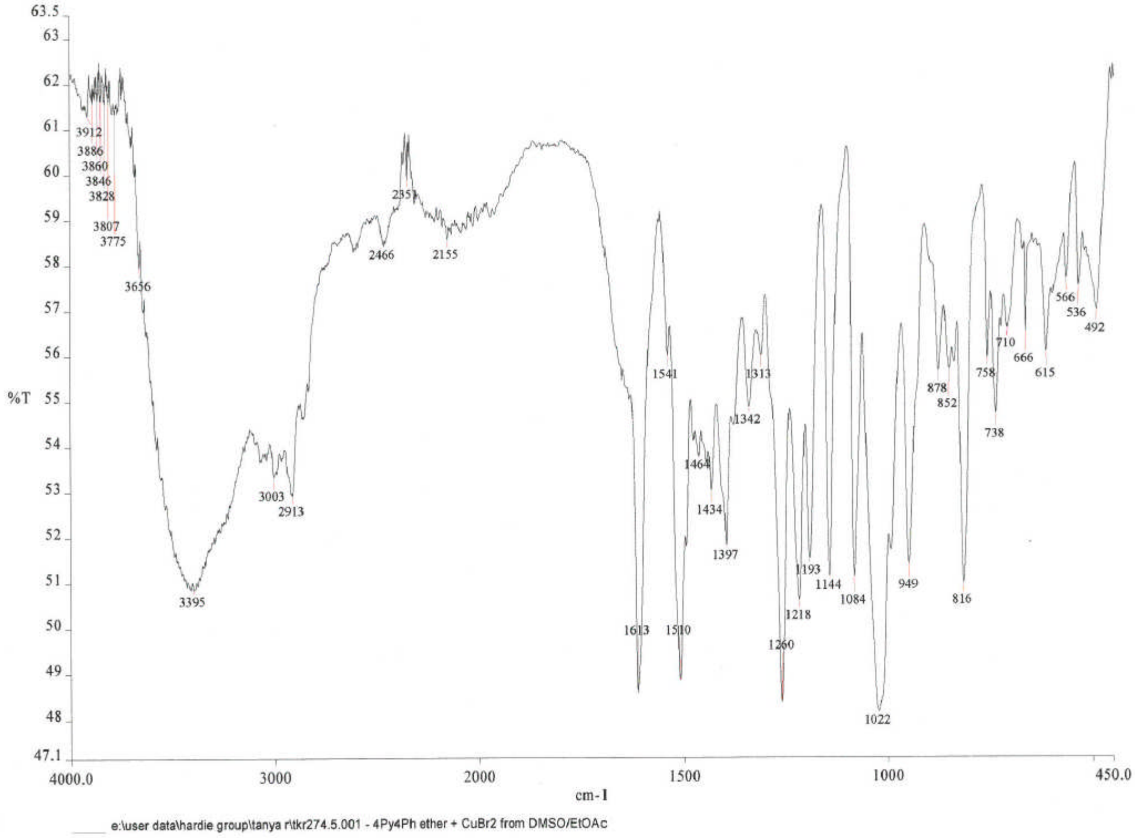

$[Cu_2(L1)_2(OTf)_2(NMP)_2(H_2O)_2]\cdot 2(OTf)\cdot 2NMP$ 4

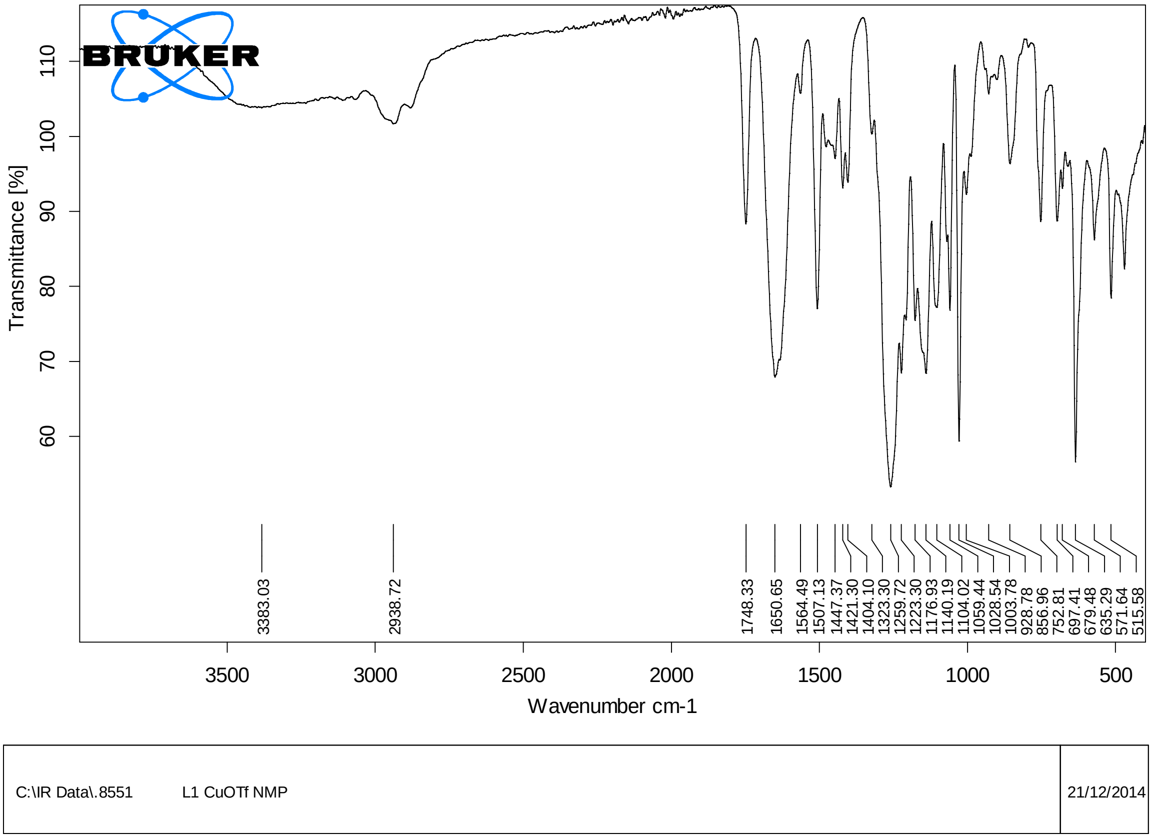

Page 1/1

[Cu(L1)(NCMe)]·BF_4·2(CH_3CN)·H_2O 5

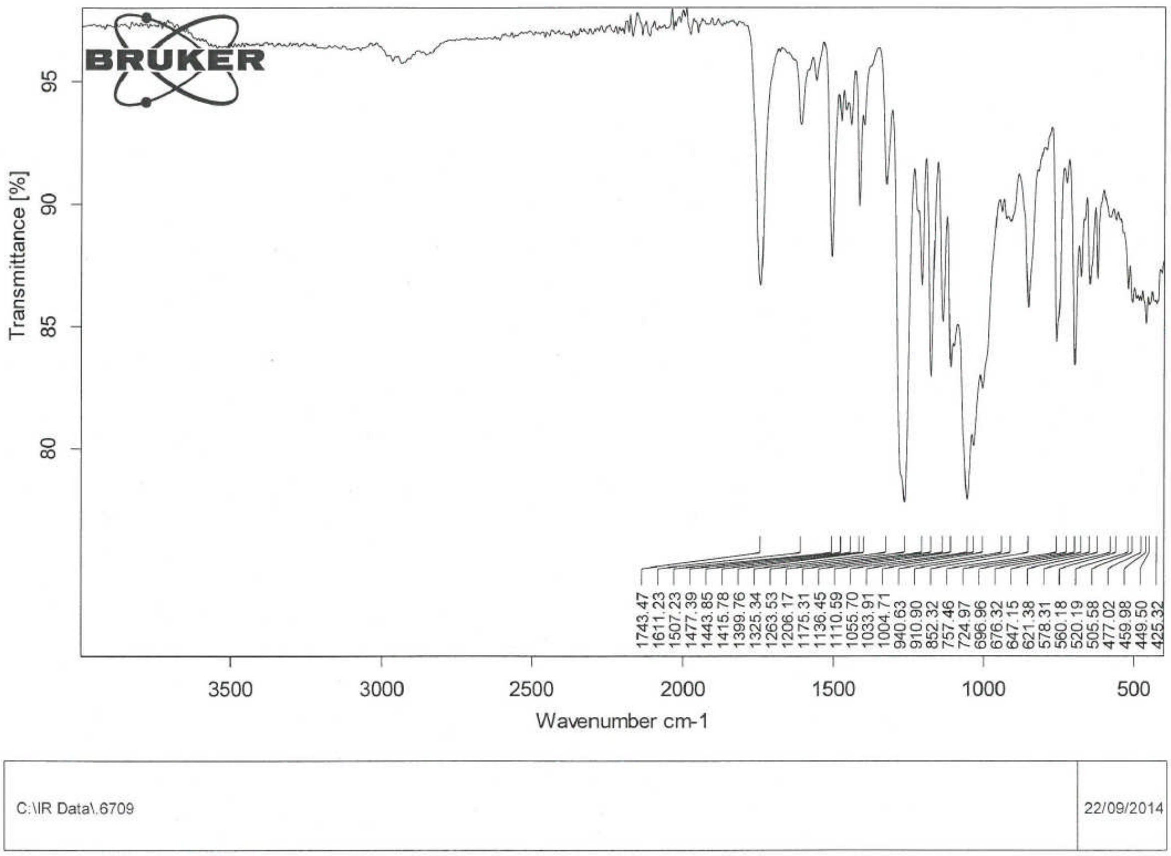

[Cu_2($L1)_2$(NMP)(H_2O)]·4BF_4·12NMP·1.5H_2O 6

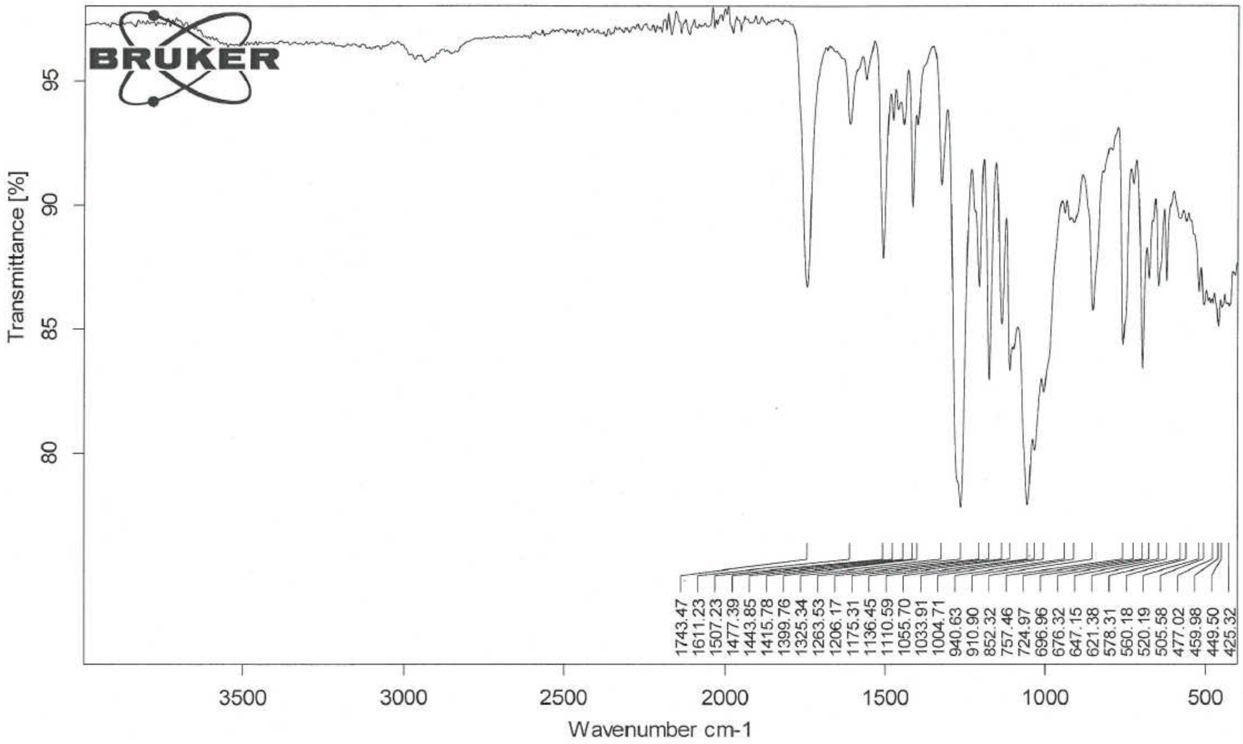

[$Cu_2(L2)_2Br_3(DMSO)$]·Br·n(DMSO) 7

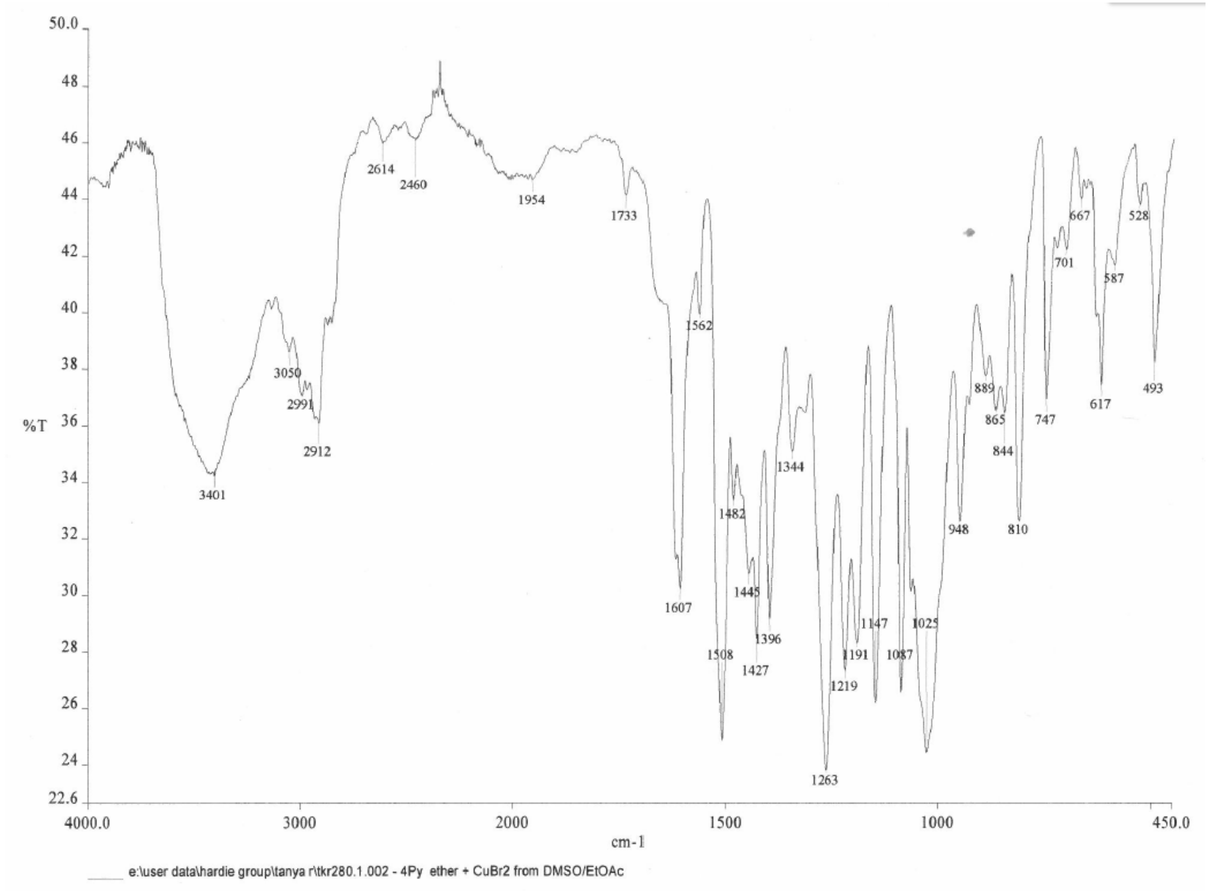

Thermal Gravimetric Analysis

$[Cu^{I}_{4}Cu^{II}_{1.5}(L1)_3(CN)_6]\cdot CN\cdot n(DMF)$ 1.

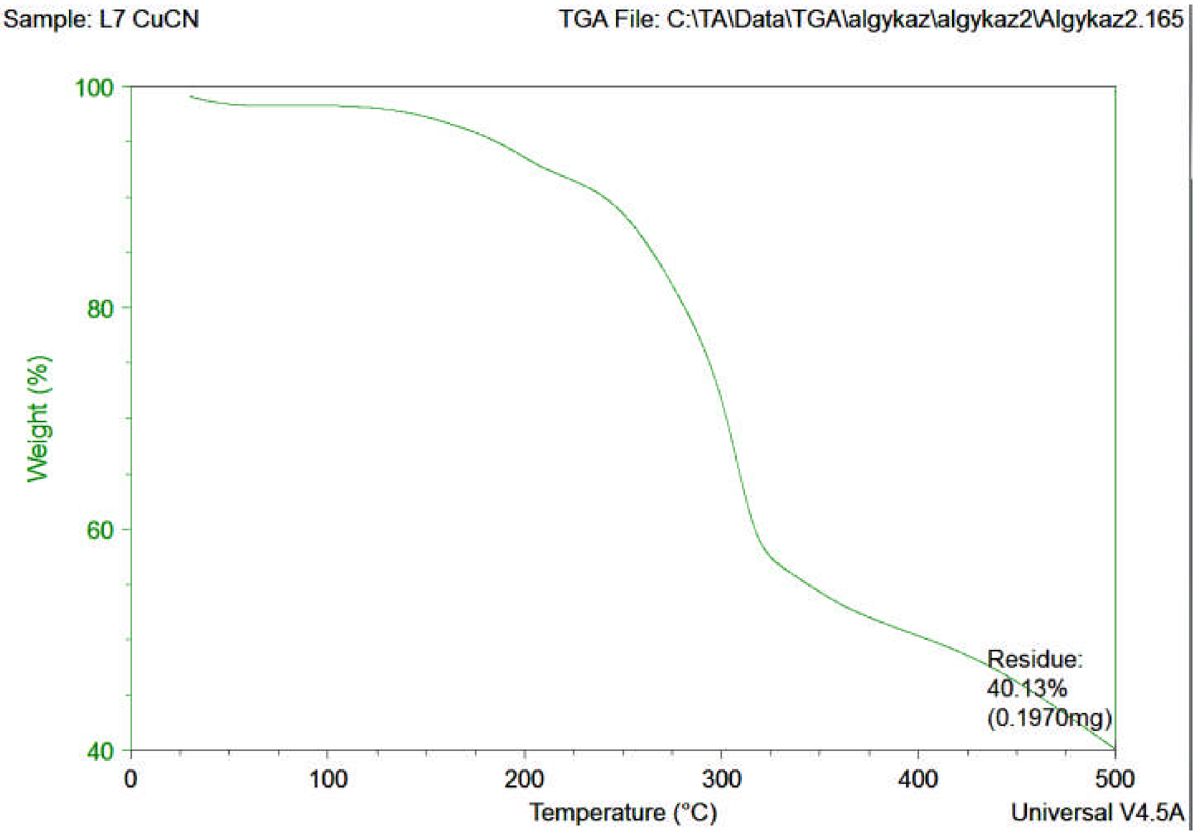

$[Cu_3(L2)_4(H_2O)_3]\cdot 6(OTf)\cdot n(DMSO)$ 2

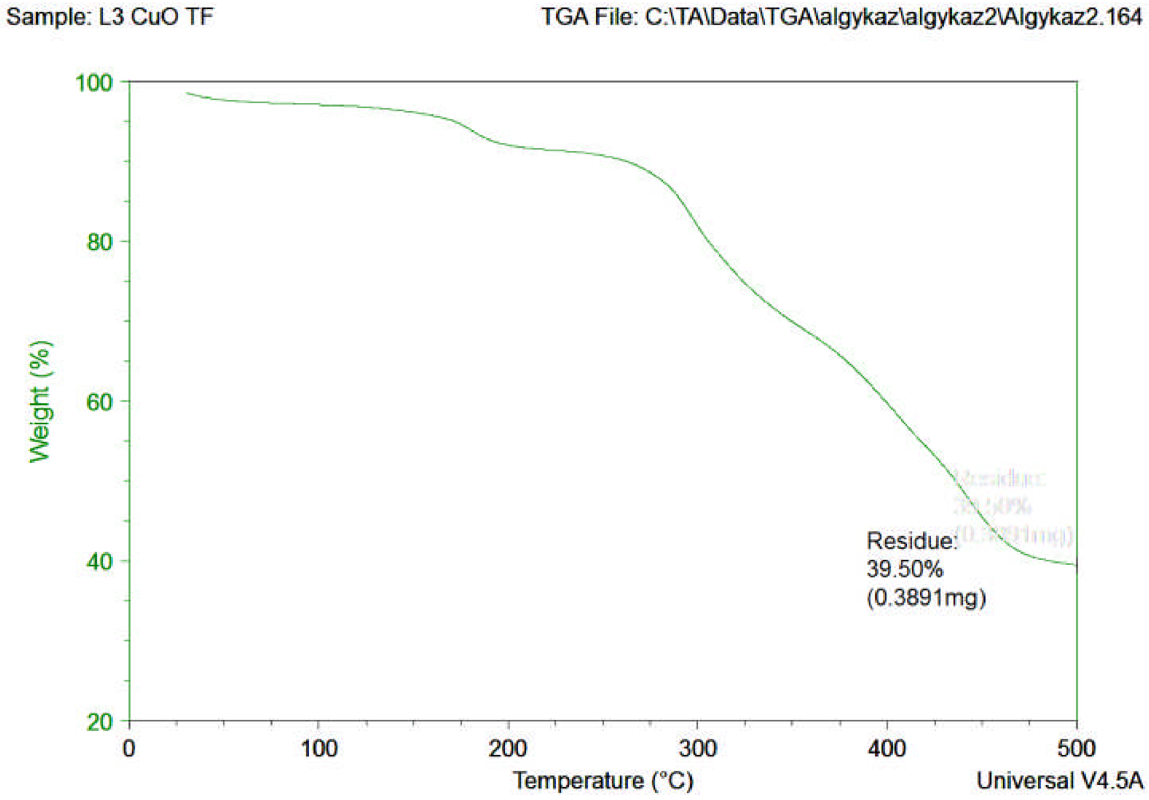

$[Cu_2(L3)_2Br_2(H_2O)(DMSO)]\cdot 2Br\cdot n(DMSO)$ 3

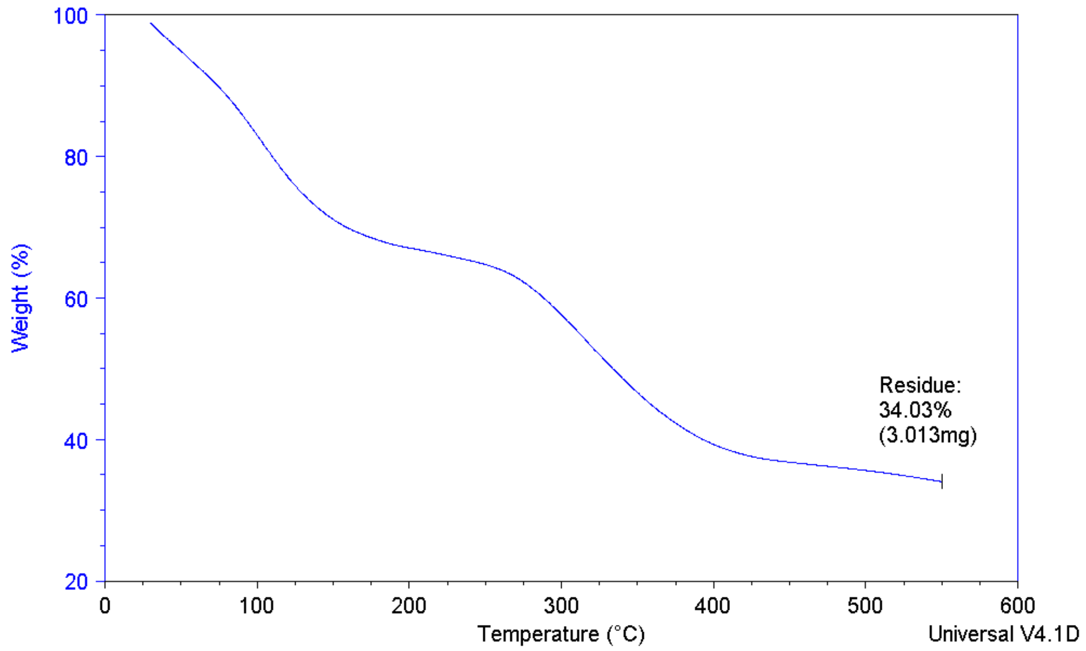

$[Cu_2(L1)_2(NMP)(H_2O)]\cdot 4BF_4\cdot 12NMP\cdot 1.5H_2O$ 6

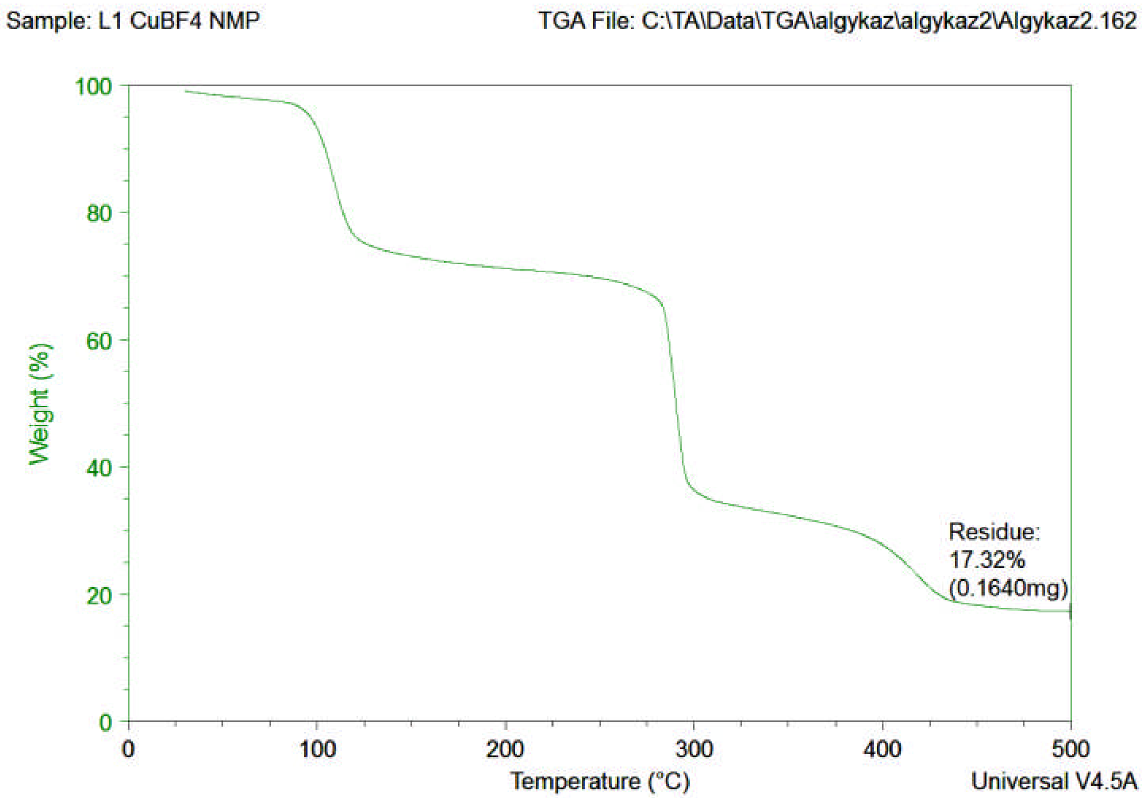

[Cu$_2$(L2)$_2$Br$_3$(DMSO)]·Br·n(DMSO) 7

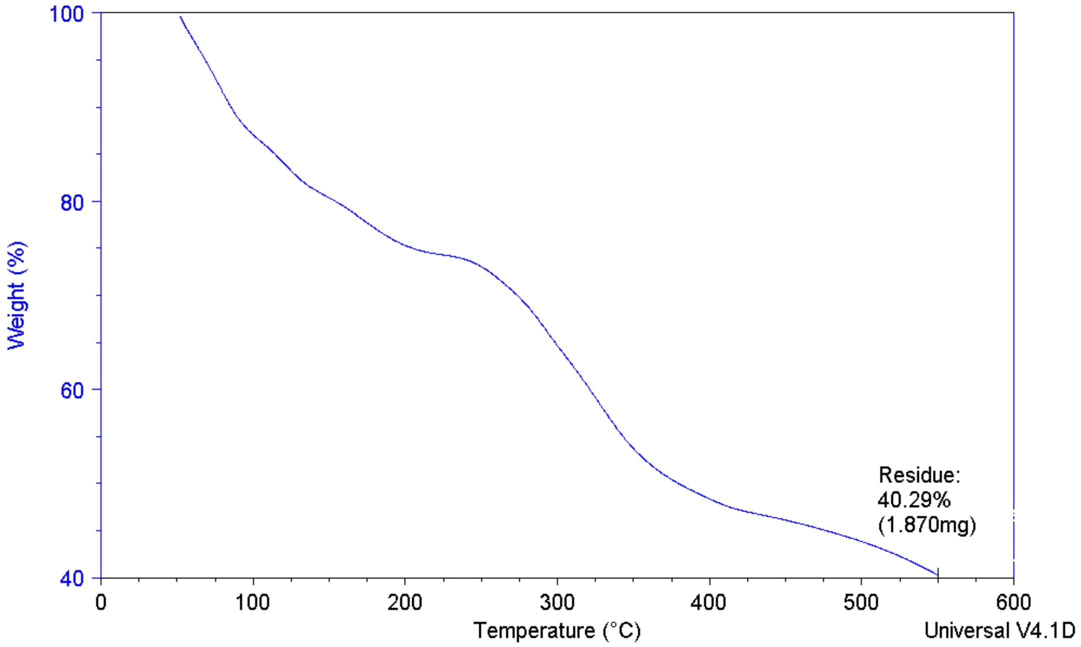

Powder XRD for bulk sample of complex [Cu(L1)(NCMe)]·BF$_4$·2(CH$_3$CN)·H$_2$O 5
(showing it is not phase pure consistent with microanalysis results)

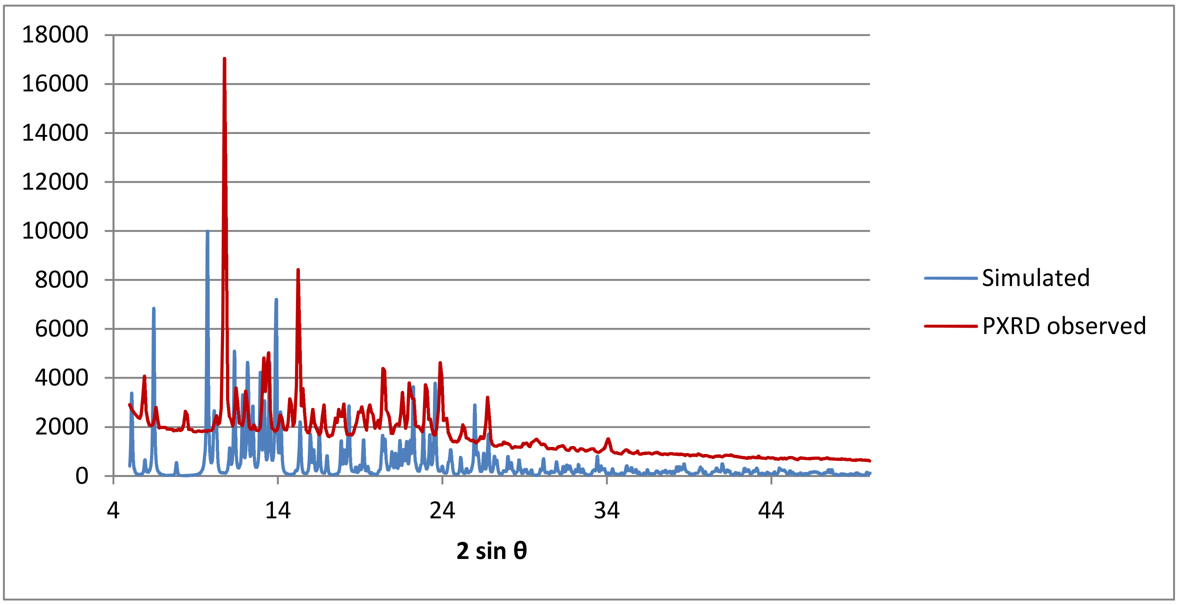

Fullerene-C_{60} sorption experiments with complex 2

Raman Spectroscopy

Raman spectroscopy was performed on a RENISHAW invia Raman Microscope with a laser power of 0.5% and a 10 second acquisition time for the vibrational range 200-2000 cm^{-1}. Multiple accumulative scans could not be collected to reduce the signal to noise ratio as decomposition of C_{60} occurred and the corresponding peaks were lost over time. Peaks at 1470, 496 (2*Ag* modes) and 272 cm^{-1} were present for the pure C_{60} sample supplied by Sigma-Aldrich, and peaks at 1470, 487 and 268 cm^{-1} were present in the polymer crystals soaked in a saturated solution of C_{60} in toluene for three weeks at room temperature, consistent with the uptake of fullerene. A small raman shift (9 cm^{-1}) was observed on binding of C_{60} within the coordination polymer material as is commonly observed due to interaction of C_{60} with the network.[4]

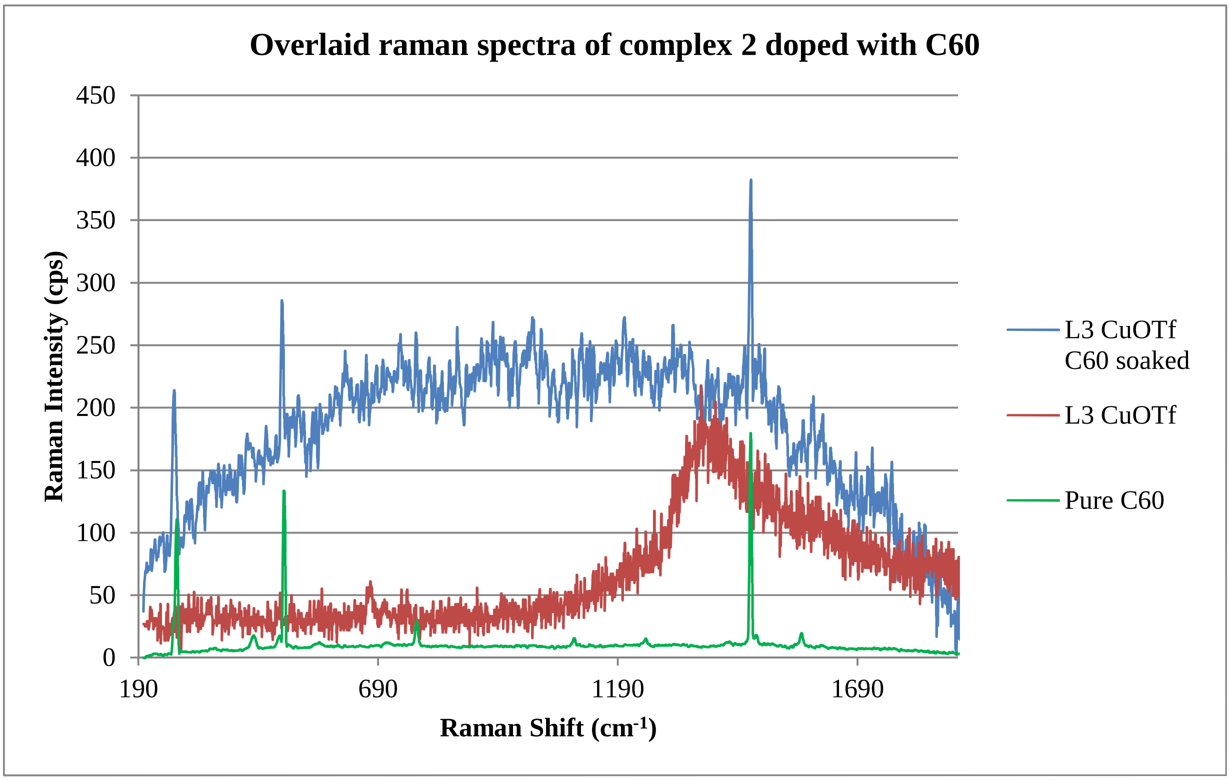

Optical microscopy images of C_{60}-uptake crystals

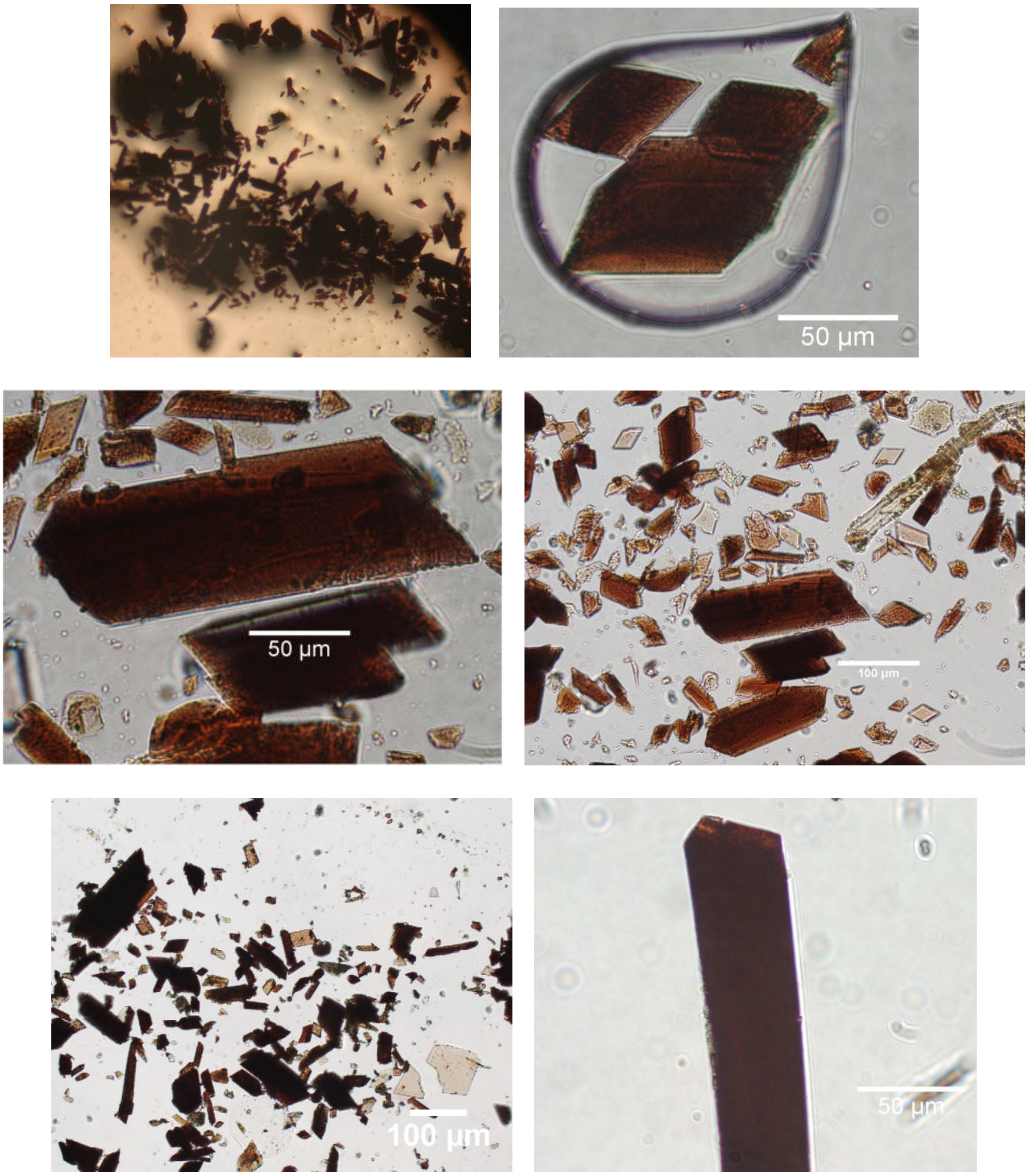

References

1. G. M. Sheldrick, *Acta Crystallogr. A* **2008**, *64*, 112.

2. P. Van der Sluis, A. L. Spek, *Acta Crystallogr. A* **1990**, *46*, 194.

3. A. L. Spek, *Acta Crystallogr. D* **2009**, *65*, 148.

4. J. Winter, H. Kuzmany, *Phys. Rev. B* **1996**, *54*, 17486; H. K. Chae, D. Y. Siberio-Pérez, J. Kim, Y.-B. Go, M. Eddaoudi, A. J. Matzger, M. O'Keeffe, O. M. Yaghi, *Nature* **2004**, *427*, 523.
